# Nanoscale Technologies in the Fight against COVID-19: From Innovative Nanomaterials to Computer-Aided Discovery of Potential Antiviral Plant-Derived Drugs

**DOI:** 10.3390/biom12081060

**Published:** 2022-07-30

**Authors:** Nunzio Iraci, Carmelo Corsaro, Salvatore V. Giofrè, Giulia Neri, Angela Maria Mezzasalma, Martina Vacalebre, Antonio Speciale, Antonina Saija, Francesco Cimino, Enza Fazio

**Affiliations:** 1 Department of Chemical, Biological, Pharmaceutical and Environmental Sciences, University of Messina, Viale F. Stagno D’Alcontres 31, I-98166 Messina, Italy; nunzio.iraci@unime.it (N.I.); sgiofre@unime.it (S.V.G.); giulia.neri@unime.it (G.N.); antonio.speciale@unime.it (A.S.); asaija@unime.it (A.S.); 2 Department of Mathematical and Computational Sciences, Physics Science and Earth Science, University of Messina, Viale F. Stagno D’Alcontres 31, I-98166 Messina, Italy; angelamaria.mezzasalma@unime.it (A.M.M.); mvacalebre@unime.it (M.V.); enfazio@unime.it (E.F.)

**Keywords:** SARS-CoV-2, nanosystems, antiviral activity, molecular docking, drug delivery systems, nanodecoys, virus spread control measures, terpenoids

## Abstract

The last few years have increasingly emphasized the need to develop new active antiviral products obtained from artificial synthesis processes using nanomaterials, but also derived from natural matrices. At the same time, advanced computational approaches have found themselves fundamental in the repurposing of active therapeutics or for reducing the very long developing phases of new drugs discovery, which represents a real limitation, especially in the case of pandemics. The first part of the review is focused on the most innovative nanomaterials promising both in the field of therapeutic agents, as well as measures to control virus spread (i.e., innovative antiviral textiles). The second part of the review aims to show how computer-aided technologies can allow us to identify, in a rapid and therefore constantly updated way, plant-derived molecules (i.e., those included in terpenoids) potentially able to efficiently interact with SARS-CoV-2 cell penetration pathways.

## 1. Introduction

The novel coronavirus, Severe Acute Respiratory Syndrome Coronavirus 2 (SARS-CoV-2), emerged from Wuhan, China in November 2019. COVID-19 (Coronavirus disease 2019) has affected all countries across the world, with more than 532 million cases and 6.3 million deaths as of June 2022 [1]. SARS-CoV-2 is primarily transmitted from person to person via respiratory droplets and aerosols produced when talking, coughing, and sneezing [2], from both symptomatic and asymptomatic people [3,4]. The mean incubation period (which is the time between exposure and development of symptoms) is around 5–7 days, but can reach 14 days [5], whereas the prevalence of people infected with SARS-CoV-2 remaining asymptomatic is estimated to be around 30–40% [6,7]. Most patients develop only mild to moderate symptoms (fever and dry cough). However, around 15% develop severe illness (dyspnea, respiratory rate ≥ 30 breaths per minute, blood oxygen saturation ≤ 93%, lung infiltrates > 50%), requiring oxygen support, and 5% become critically ill with respiratory failure, acute respiratory distress syndrome (ARDS), sepsis, thromboembolism, and multiorgan failure [8]. Clinical symptoms are more severe in older patients and in those with comorbidities, including hypertension, diabetes, cardiovascular diseases, respiratory diseases, cancer, obesity, and renal diseases [9]. Apart from the respiratory symptoms, SARS-CoV-2 may cause cardiovascular, neurological, and gastrointestinal symptoms. Hypercoagulability is a common and early manifestation of coagulopathy, and a predictor of disease worsening, with thromboinflammation playing a crucial role in severity and mortality of COVID-19 [10]. It became evident early on that severe COVID-19 is a systemic hyperinflammatory disease derived from the so-called cytokine storm, a term describing a condition of uncontrolled systemic hyperinflammation triggered by the release of a big amount of pro-inflammatory cytokines, such as IL-1, IL-6, IL-18, IFN-γ, and TNF-α, among others [11]. To date, nanotechnological platforms have been explored for their employment in diseases due to several viruses [12], and for the development of new effective therapeutic treatments as well as diagnostic tools, innovative materials, and drugs useful for infection prevention and control [13,14,15].

Strategies involving nanodrugs for the treatment and prevention of viral diseases, including COVID-19, are briefly described below:

**Trapping effects:** the first step in a viral invasion is its attachment to the host cell membrane. Therefore, it is highly considered when designing anti-infection nanomaterials, including those based on cell membrane properties; in fact, cell-mimicking nanoparticles (NPs), displaying a pathogen’s cognate receptor on their surface, represent an emerging class of therapeutics that are potentially effective against SARS-CoV-2 and other viruses [16].

**Inhibition of viral entry:** the inhibitory effect of nanodrugs on cell entry by viruses and related mechanisms has been studied extensively. Viral attachment and entry usually requires interaction between viral surface protein(s) and receptor(s) on host cell membranes; thus, nanomaterials that interfere with such interactions are promising antiviral agents which can act at a relatively accessible extracellular level, and thus prevent the infection state at an early different stage [17]. Some nanodrugs interrupt virus–cell interactions by blocking viral surface proteins and cell membrane receptors. In particular, as better described in the paragraphs below, SARS-CoV-2 uses the viral Spike protein and the host cell angiotensin-converting enzyme II (ACE2) protein, as the main method of cell penetration, through a “Lock and Key” mechanism that allows the virus access to cells that have the corresponding lock. These sites can be found throughout the body, but especially in the lungs, heart, and arteries. This process emphasizes the need to find a treatment that could potentially inhibit this (and similar) “Lock and Key” process(es) altogether.

**Inhibition of viral replication:** nanodrugs can inhibit viral replication by interacting with the viral protein/genome or inducing a suppressive environment for intracellular viral replication. For example, relevant antiviral activity against herpes simplex virus type 1 (HSV-1) has been shown by copper (II) oxide (CuO) NPs via degradation of the viral genome or oxidation of viral proteins [18]. Similarly, zinc oxide (ZnO) NPs act against H1N1 influenza virus infection when added after viral infection, highlighting that the inhibition mechanism takes place after viral entry [19].

**Viral inactivation effects:** nanodrugs, including silver (Ag) and titanium (Ti) complexed with biomolecules, and carbon-based nanomaterials, take directly contact with viruses and induce viral inactivation by different mechanisms depending on the nanomaterial and virus. The destruction of the viral envelope or capsid (for example by photocatalytic oxidation) is a common mechanism by which nanomaterials induce the inactivation of viruses. For example, Yang et al. reported the ability of Ag NPs modified with curcumin to inactivate the viral envelope glycoproteins of respiratory syncytial virus [20], while Gao et al. demonstrated that 3,6-sulfated chitosan is able to bind viral capsid proteins of human papillomavirus blocking the virus absorption process [21].

**Figure 1:**
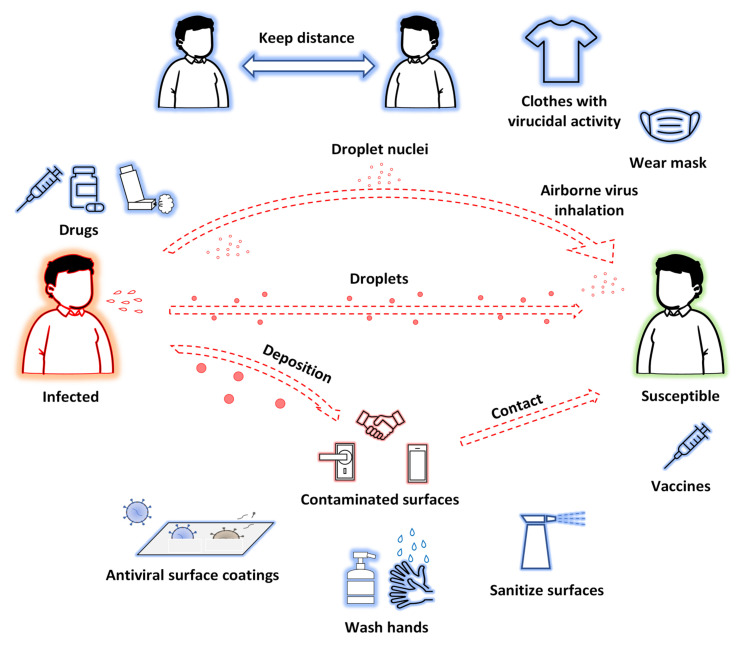
Transmission methods of SARS-CoV-2 and possible approaches to prevent and treat COVID-19.

Transmission methods of SARS-CoV-2 and possible approaches to prevent and treat COVID-19 are shown in Figure 1.

The first issue to be underlined to explain nanotechnologies’ great potential for improving prevention and treatment of viral diseases is that nanomaterials [16] are flexibly functionalized with specific molecules at precise targets. Therefore, they can inhibit viral infection from multiple directions, may present broad-spectrum antiviral properties due to common underlying mechanisms, and are sometimes more affordable in comparison with other antiviral approaches (for example, antibodies). In fact, the unique properties of nanomaterials—particularly their small size (1–100 nm), but also their high surface-to-volume ratios and modifiable surfaces—are beneficial for contact with viruses and contribute to multiple antiviral effects. Nanomaterials have been reported to suppress cell entry and viral replication; moreover, their numerous surface binding sites facilitate interactions with target molecules, consequently trapping and inactivating viruses. So, it is evident that modern nanotechnologies do not solely aim to discover new antiviral therapeutics (or to repurpose known products). Nanocomposites can be grouped with different antimicrobial agents in order to obtain a synergistic effect, such as that achieved with chitosan, ZnO, and Ag NPs [22]. Furthermore, nanomaterials may be used to produce innovative drug delivery systems (DDSs) to improve the pharmacokinetic properties of loaded drugs. If drugs are sensible to external stimuli, they may be projected as endowed with auxiliary functions useful for inactivating viruses or mimicking host cells, or they could be functionalized with specific ligands that are able to bind molecular components of the target, and so on. In this way, there is a strong effort toward the application of inhalation therapy that takes advantage of nasal spray anti-inflammatory activity [23,24].

**Figure 2:**
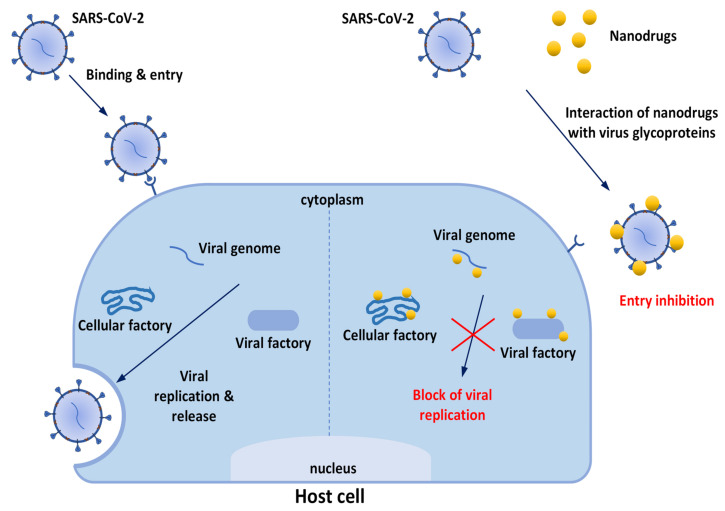
Possible mechanisms involved in the effectiveness of nanodrugs for prevention and treatment of COVID-19.

Figure 2 reports the possible mechanisms involved in the effectiveness of nanodrugs for prevention and treatment of COVID-19. The discovery and development of antiviral nanodrugs take enormous advantages from the employment of in silico and computational approaches, which are widely used today and strongly contribute to identification of the potential specific interactions between the virus’ macromolecules and the investigated antiviral systems [25,26,27,28]. There is also a growing interest in the design of coordination compounds to be used against COVID-19 infections [29,30]. Advanced computational techniques are fundamental in discovering or repurposing active therapeutics, obtained via chemical synthesis but also derived from natural (particularly vegetable) matrices. Indeed, today, a high percentage of approved therapeutic molecules are natural products [31]. Plant-derived products have always been rich resources for discovery of drug/dietary supplements that are useful against several diseases; in particular, a relevant number of natural products possess promising antiviral effects against human CoVs [32] and may guide the development of novel anti-COVID-19 drugs. Several natural products have been shown to be effective in other coronavirus illnesses, such as SARS and MERS (Middle-East Respiratory Syndrome), evidencing the possibility of an efficacious employment in this new pandemic (see Refs. [33,34,35]). Among others, Ag and Fe${}_{3}$O${}_{4}$ NPs have been successfully used in synergy with plant extracts to enhance antibacterial and antiviral properties [36,37,38,39].

On the other hand, developing a new drug is a very long process, and this represents a problem, especially in the case of pandemics, where an effective therapy is required as soon as possible. Besides the repurposing of known and approved drugs, plant sources are also an excellent strategy for finding new and low-cost drugs in a short time because they are endowed not only with favorable efficacy, but also tolerable toxicity. Finally, natural products have been used for many years to treat viral infections thanks to several bioproperties, including anti-inflammatory and immunomodulatory activities, which are suitable for treatment of COVID-19 patients. A particular issue to be underlined is that to defeat COVID-19 (although this can be generalized to other transmissible viral and bacterial infectious diseases), in addition to therapeutic treatments, control measures are essential. The recent outbreak of COVID-19 has demonstrated that the adoption of passive measures helps minimize the impact of current and future infection outbreaks, so that innovative nanomaterials may have a main role in the development of virus spread control measures which would be efficient against SARS-CoV-2. For example, research on antiviral textiles (made or covered with nanomaterials) has received considerable attention, since these can effectively inhibit the spread of viruses or the formation of biofilms on their surface, reducing the risk of infection/re-infection [40,41,42]. In 2019, the global daily output of medical masks reached 40 million pieces, generating more than 15,000 tons of daily waste; the daily output of masks increased enormously in 2020 (more than 20 times in comparison with 2019) [43,44]. When COVID-19 patients cough or sneeze, tiny virus-containing droplets are emitted and can contaminate surrounding surfaces, contributing to virus spread. Then, the SARS-CoV-2 can remain stable for several hours, depending on the physicochemical characteristics of these surfaces. This has increased researchers’ interest in developing not only new hand and surface disinfectants, but also self-decontaminating surface materials [45].

The aim of our paper is to provide, two years after the beginning of the SARS-CoV-2 pandemic, an updated overview of the state of the art on the usefulness of nanoscale technologies in coping with this global health crisis. So, the first part of the review is focused on the most recent and innovative nanomaterials that have been projected and proven to be effective against SARS-CoV-2, as well as possibly against other viruses, both in the field of therapeutic agents and of measures to control virus spread. The second part of the review aims to show how computer-aided technologies can allow us to identify, in a rapid and therefore constantly updated way, plant-derived molecules that are potentially able to efficiently interact with SARS-CoV-2 cell penetration pathways, taking the terpenoids class as an example. This class of plant compounds, known for their wide-spectrum antiviral properties, includes some molecules recently reported as potentially active against SARS-CoV-2 [46,47].

The present narrative review was conducted using Pubmed and Web of Science, covering the interval between January 2020 and April 2022; further articles were identified by examining reference lists in the papers screened and in reviews recently published on this topic. Relevant original papers were selected by reading their titles, abstracts, and full text. For the first part of the review, papers were searched using a combination of the terms “COVID, coronavirus, viruses, nanotechnologies, nanomaterials, nanodrugs, nanoparticles, metals, nanosystems”, and were included in the review if reporting data significantly related to antiviral activity against SARS-CoV-2 or treatment of COVID-19. As to its second part, papers were searched using a combination of the terms “COVID, coronavirus, terpenes, terpenoids, herbal, plants, molecular docking, molecule dynamic analysis, computer-aided technologies, ACE2, spike, GRP78”, and were included if reporting original data about the chemical interaction of terpenoids with proteins relevant for virus penetration, particularly ACE2 and/or Spike. This second section of the review was completed by original in silico data, obtained in our facilities, evidencing the interaction of some promising terpenoids with the host GRP78 protein, more recently identified as a receptor for SARS-CoV-2 S protein.

## 2. SARS-CoV-2 and COVID-19

**Figure 3:**
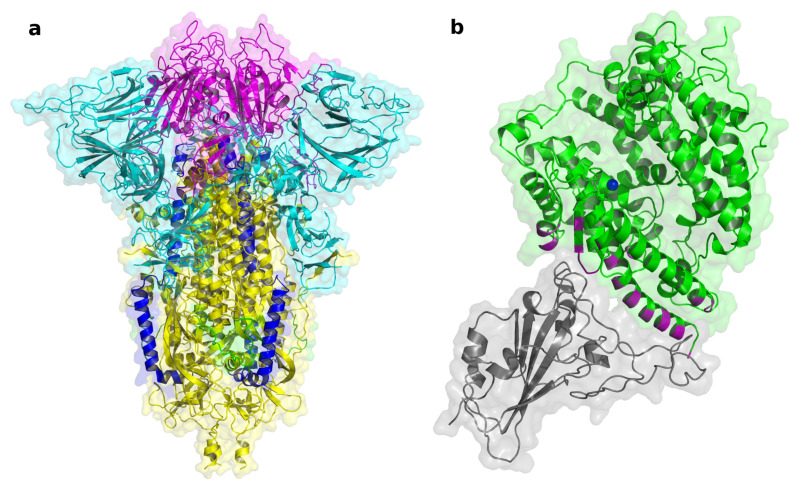
(**a**) Cryo-electron microscopy structure of SARS-CoV-2 spike protein (residues 1-1147—PDB ID: 7DCC). Spike protein domains are color coded as follows: S1—cyan; RBD—magenta; S2—yellow; FP—green; HR1—blue [53]. (**b**) X-ray crystal structure of ACE2 bound to SARS-CoV-2 spike protein (PDB ID: 6M0J). ACE2 is depicted in green cartoons, with the spike/ACE2 interface residues highlighted in magenta and the zinc atom represented by a blue sphere. Spike protein RBD is depicted in gray [54].

SARS-CoV-2 belongs to the β coronavirus subgroup of the Coronaviridae family, order Nidovirales. The CoVs are a highly diverse group of enveloped, positive-sense, single-stranded RNA (+ssRNA) viruses [48], infecting humans, other mammals, and avian species. SARS-CoV-2 entry into the host cell is mediated by the binding of the surface unit, S1, of the spike (S) protein, to the cellular surface protein Angiotensin-Converting Enzyme 2 (ACE2), which facilitates viral attachment to the surface of the target cell. The Receptor Binding Domain (RBD) located on S1 contains a motif that binds to the N-terminal extracellular peptidase domain of ACE2, resulting in a SARS-CoV-2/ACE2 complex [49,50]. In addition, cell entry requires S protein priming by the cellular Transmembrane protease, serine 2 (TMPRSS2), leading to S protein cleavage into two subunits, S1 (containing the RBD) and a fusion fragment (S2), which facilitates viral fusion with the membrane of the target cell. The S1 subunit contains an N-terminal domain and the RBD; in the S2 subunit are the fusion peptide (FP), heptapeptide repeat sequences 1 (HR1) and 2 (HR2), the TM domain, and the cytoplasm domain (Figure 3). In case of low levels of TMPRSS2 on the target cell, the virus is internalized via endocytosis and, after endosomal acidification, S2 is cleaved by the enzymes cathepsins [51]. Finally, regardless of which of the two mechanisms was followed, the viral RNA is released into the cytoplasm of the host cell [52].

The host receptor ACE2 is a Zn-dependent monocarboxypeptidase and a member of the renin–angiotensin system (RAS). It is widely expressed in several organs, such as the heart, kidneys, testis, brain, upper airways, lungs, gut, and liver, and plays a vital role in cardiovascular and renal homeostasis. In fact, ACE2 converts angiotensin II into the vasodilator angiotensin 1–7 to counterbalance the vasoconstrictor role of ACE in the RAS, and thus controls hypertension and sodium–water retention, and protects multiple organs, including the heart, kidneys, and lungs [55]. Currently, one of the most effective antiviral mechanisms is to block or interfere with the interaction between viral S protein and human ACE2.

Emerging evidence demonstrates interactions between S protein and other host receptors and proteins, apart from ACE2, that then may represent alternative routes for viral entry. Among these are Glucose Regulated Protein 78 (GRP78), CD147, and innate immune system proteins, such as C-lectin type receptors (CLRs) and toll-like receptors (TLRs) [56,57]. GRP78 is a chaperone protein controlling the unfolded protein. It is normally localized into the lumen of the endoplasmic reticulum bound to inactivating enzymes. Accumulation of unfolded or misfolded proteins results in GRP78 release and translocation to the plasma membrane [58]. Patients infected with SARS-CoV-2 have increased gene expression and serum concentrations of GRP78 [59] and, as it is able to bind to the S protein of SARS-CoV-2 [60] and, as previously reported, is involved in the entry of some viruses [61], it may represent another potential therapeutic target in COVID-19 treatment.

**Figure 4:**
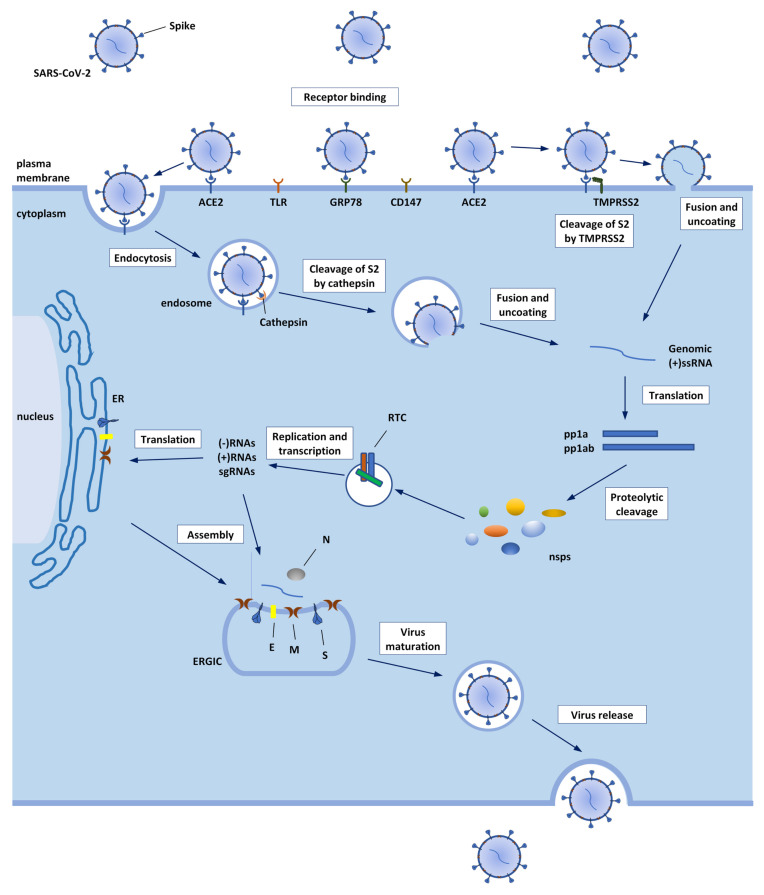
**SARS-CoV-2 life cycle**. SARS-CoV-2 entry into the host cell is mediated by the binding of the Spike protein (S) to ACE2. Then, entry requires S protein priming by the TMPRSS2, leading to S protein cleavage, which facilitates viral fusion with the membrane of the target cell. In case of low levels of TMPRSS2 on the target cell, the virus is internalized via endocytosis and, after endosomal acidification, S2 is cleaved by the enzymes cathepsins. Then, regardless of which of the two mechanisms was followed, the viral RNA is released into the cytoplasm of the host cell. Emerging evidence supports interactions between S protein and other host receptors and proteins (such as GRP78, CD147, TLRs), apart from ACE2, that may represent alternative routes for viral entry. After the release of viral (+)ssRNA genome into the cytoplasm, pp1a and pp1ab are produced by cellular ribosomes and then processed by viral proteases (PLpro and 3CLpro) into 16 nsps that form the replicase–transcriptase complex (RTC). The RTC mediates the synthesis of (-)RNA. A full-length (-)RNA copy serves as a template for the full-length (+)ssRNA genome, whereas sgRNAs are translated into structural (S, M, E, N) and accessory proteins. The viral nucleocapsid is assembled from newly synthesized viral genomic (+)ssRNA and N proteins in the cytoplasm, and then buds into the ER–Golgi intermediate cavity (ERGIC) reaching the S, E, and M for viral assembly. The new virions are then released from the cells via exocytosis.

The genome sequence of SARS-CoV-2 consists of approximately 29,903 nucleotides and contains 14 open reading frames (ORFs). After the release of viral (+)ssRNA genome into the cytoplasm, ORF1a and ORF1ab, located in the first two-thirds of the genome, are directly translated by cellular ribosomes into the two polyproteins pp1a and pp1ab, which are then processed by two viral proteases, papain-like protease (PLpro) and 3-chymotrypsin-like protease (3CLpro, or main protease), into 16 non-structural proteins (nsps) and form the replicase–transcriptase complex (RTC), which contains the RNA-dependent RNA polymerase (RdRp, nsp12) in its core. In vesicles originating from the endoplasmic reticulum (ER), the RTC mediates the synthesis of (-)RNA. A full-length (-)RNA copy serves as a template for the full-length (+)ssRNA genome, whereas subgenomic RNAs (sgRNAs) are translated into the structural proteins, which are spike (S), membrane (M), envelope (E), and nucleocapsid (N), as well as several accessory proteins, all encoded by the last third of the genome [62]. The viral nucleocapsid is assembled from newly synthesized viral genomic (+)ssRNA and N proteins in the cytoplasm, and then buds into the ER–Golgi intermediate cavity (ERGIC), reaching the S, E, and M for viral assembly. The new virions are then released from the cells via exocytosis [63,64]. The SARS-CoV-2 life cycle is shown in Figure 4.

SARS-CoV-2 shows a continuous evolution due to changes in its genetic code, and multiple variants of this virus have been discovered in the world during this pandemic. A SARS-CoV-2 variant differs from others due to one or more mutations, while a lineage is a genetically closely related group of virus variants derived from a common ancestor. A group of variants with similar genetic changes may be designated by public health organizations as a Variant of Concern (VOC), Variant of Interest (VOI), and Variant Being Monitored (VBM), if they share characteristics that require public health action. Many of these variants are mutations concerning the Spike protein, and therefore, due to the role of this protein in host cell penetration, are the main target of surveillance.

As of 15 July 2022, the European Centre for Disease Prevention and Control (ECDC) has classified Omicron (BA.1, BA.2, BA.4, and BA.5 lineages) as a VOC, due to potential increased transmissibility and reduction in neutralization by some monoclonal antibody treatments and post-vaccination sera; while Omicron BA.2+L452X and BA.2.75 are classified as VOI. The updated list of Spike mutations of interest (including changes to spike protein residues 319–541 (RBD) and 613–705 (the S1 part of the S1/S2 junction and a small stretch on the S2 side), and additional unusual changes specific to the variant) can be found at https://www.ecdc.europa.eu/en/covid-19/variants-concern (accessed on 4 June 2022). Other variants such as Delta have been descaled, especially because they are no longer circulating or do not have impact on the overall epidemiological situation.

Initially, in the early stages of the pandemic, repositioning of various drugs was theorized and tested. Among these drugs are chloroquine, hydroxychloroquine, and ivermectin. Although initial studies showed positive results, subsequent trials did not confirm their efficacy and raised doubts about possible adverse effects [65,66,67,68].

From the beginning, it also became clear that dysregulation of immune responses against SARS-CoV-2 is one of the main features of COVID-19 pathogenesis; thus, corticosteroids (mainly dexamethasone) are used as immunomodulatory agents, and furthermore, interleukin (IL)-6 antagonists (tocilizumab) and Janus kinases inhibitors (baricitinib and tofacitinib) were also subsequently introduced [69,70,71,72,73]. Other agents are now gradually introduced, such as antivirals and monoclonal antibodies (mAbs), and show usefulness in some patient groups.

In addition, supportive therapy involves, in some cases, prophylaxis with antibiotics and anticoagulants. Routine use of empiric antibiotics in patients with confirmed COVID-19 is not recommended, in order to avoid the spread of antimicrobial resistance, unless evidence of bacterial superinfection is present [74]. Since thrombosis and coagulopathy seem to play an important role in SARS-CoV-2 pathogenesis [75], prophylaxis with anticoagulants is required in some cases. Non-hospitalized patients with COVID-19 should not receive prophylactic anticoagulants or antiplatelet therapy without a specific indication, whereas their use may be recommended based on illness severity [76].

Various antivirals agents have been developed for the treatment of COVID-19. Remdesivir, the first to be approved, once activated into triphosphate, competes with the natural substrate adenosine triphosphate for incorporation via RdRp into viral RNA, leading to inhibition of viral replication by terminating RNA transcription prematurely [77]. It is approved by the FDA and EMA for the treatment of mild to moderate COVID-19 in high-risk outpatients and for hospitalized patients with COVID-19 [78,79]. Some newly approved oral antivirals are paxlovid and molnupiravir. The first is a combination of the 3CLpro inhibitor nirmatrelvir and ritonavir as pharmacokinetic booster [80], whereas molnupiravir is a nucleoside analogue that, once activated, induces errors in the viral RNA as it replicates [81].

Various neutralizing mAbs, directed against the SARS-CoV-2 S protein and designed to block viral attachment and entry into human cells, thus neutralizing the virus and potentially preventing and treating COVID-19, have been approved for the treatment of COVID-19. Bamlanivimab, as well as the combination bamlanivimab/etesevimab, are no longer recommended due to reduced efficacy against variants [82]. Casirivimab and imdevimab are recombinant human mAbs that bind to non-overlapping epitopes of the S protein RBD, and thereby block binding to the human ACE2 receptor. The combination is approved for mild to moderate COVID-19 patients who are at high risk of progressing to severe disease and/or hospitalization, and to prevent infection in high-risk patients, although distribution has been paused by the US FDA because of reduced activities against the B.1.1.529 (Omicron) variant of concern [82,83]. Sotrovimab targets an epitope in the RBD of the S protein and is expected to retain efficacy against the Omicron variant. It is recommended for the treatment of non-hospitalized patients with mild to moderate COVID-19 who are at high risk of clinical progression [82,84]. Finally, Evusheld is a long-acting combination of two mAbs (tixagevimab and cilgavimab) designed to target the S protein at two different sites. The FDA has issued an Emergency Use Authorization (EUA) for individuals who are at risk of an inadequate immune response to COVID-19 vaccination or have a documented history of severe adverse reaction to an available COVID-19 vaccine or any of its components [82].

## 3. Nanomaterials for COVID-19 Prevention and Treatment

### 3.1. Metal-Based Nanomaterials

Several viruses, including SARS-CoV-2, may be successfully treated with metals (in particular, noble metals) and their complexes. As reported in the literature, biomaterials based on metals such as Ag, Cu, gold (Au), Zn, Fe, and Ti are alternatives to the currently used chemical disinfectants, with unique antiviral activities, durability, and efficacy at low concentrations. They are characterized by large-spectrum usage, and can successfully overcome the limitations faced by other conventional medicines. Indeed, their action is independent of age and comorbidity, no drug resistance is developed, and low cytotoxicity is found [85].

Despite the evidence of a biocidal activity of metals, occurring even at low concentrations [86], their antiviral mechanisms are still not completely clear because selective interactions between metals and viral macromolecules, particularly proteins, cannot be easily identified. Experimental data have shown two main antiviral performances of metals: (i) the ability to prevent the viral infection inhibiting the entrance of virus within the host system; (ii) the ability to affect processes involved in virus replication [87].

Thanks to the ability to slowly release the metal ions from metal NPs, metal-based materials are recently employed as virus spread control tools, providing long-term protection against viruses. For example, it was demonstrated that the use of these materials as coating agents can strongly reduce virus infectivity for several weeks [88]. In a liquid environment, the transport of the virus particles is much slower compared to that of metal ions from metal NPs, and thus, considering the relative diffusion coefficients (Stokes–Einstein equation), the antiviral effect from metal ions occurs faster [89].

#### 3.1.1. Silver-Based Nanomaterials

Ag NPs, thanks to their tunable physicochemical properties, easy production routes, and remarkable biological effects, including excellent antimicrobial action, are one of the most investigated nanomaterials for biomedicine applications [90]. The recent need for an effective agent against SARS-CoV-2 has led to increasing attention being paid to the Ag NPs’ antiviral activity. As is well known, it is correlated with several mechanisms, including interactions with viral envelope and with viral surface proteins (preferentially towards the ones rich in sulfhydryl groups, leading to disulfide bond cleavage, which destabilizes the protein structure). Other mechanisms regard the interactions with host cell pathways to prevent virus penetration and the interactions with viral factors necessary for virus replication [91,92].

To better understand the mechanism of the antiviral action of Ag NPs against SARS-CoV-2, Rodrigues et al. [93] investigated, through a computational approach, the spontaneous interaction among silver ions (Ag${}^{+}$) and five amino acids (glutamate, isoleucine, leucine, threonine, and lysine) present in the structure of the SARS-CoV-2 spike protein. The theoretical studies demonstrated that the interactions between Ag${}^{+}$ and -NH${}_{2}$ groups are more favorable than those with -C=O. The negative values of Gibbs free energies and the negative enthalpy energy variation (ΔH<0) indicate that the Ag${}^{+}$–amino acids interactions are spontaneous and exothermic, highlighting the potentiality of Ag NPs to fight SARS-CoV-2. Jeremiah et al. evaluated the efficiency of Ag NPs with different diameters necked or capped with polyvinylpyrrolidone, against SARS-CoV-2 infection. It was demonstrated that Ag NPs, with a diameter from 2 to 15 nm at concentrations between 1 ppm and 10 ppm, are able to prevent the entry of SARS-CoV-2 into VeroE6/TMPRSS2 cells by disrupting viral integrity, whereas they display cytotoxic effects at a concentration of 20 ppm or higher [94]. The findings obtained on cells exposed to virus have allowed us to hypothesize that Ag NPs interactions with ACE2 receptors and/or intracellular mechanisms may be involved in the observed effects. This material is proposed for us on inanimate and non-biological surfaces as a virus spread control measure, due to its cytotoxicity.

Merkl et al. have tested the antiviral activity against SARS-CoV-2 of glass and porous filter media (glass fiber filters and FFP3 filters) coated with Ag, CuO, and ZnO [89]. Comparing the performance of the three metallic nanostructures, Ag exhibited the highest antiviral activity against SARS-CoV-2, reducing the viral load up to 75% after 5 min and 98% after 120 min. Using CuO nanostructured film, the viral load was reduced by 54% and 76% after 30 and 120 min, respectively, whereas the virus stability remained almost unchanged with ZnO nanostructured film. The different results are ascribed to different mechanisms: Ag nanomaterial has a direct antiviral activity releasing Ag${}^{+}$ ions, which inhibit viruses by binding to viral proteins or directly lysing membranes, while CuO has indirect antiviral action, inducing the generation of reactive oxygen species (ROS).

#### 3.1.2. Gold-Based Nanomaterials

Thanks to their good biocompatibility, poor immunogenicity, and ability to bind biological ligands, Au NPs are interesting candidates as antiviral agents [95]. Au metallodrugs are proposed as effective inhibitors of SARS-CoV-2, and their antiviral mechanism is based on two main pathways: the first regards the ability of Au NPs to prevent virus binding to cell membranes, and the second is based on the capability of Au NPs to inhibit virus proliferation. In this way, Rothan et al. investigated the antiviral activity of Auranofin against SARS-CoV-2 [96]. Auranofin is a gold-containing triethyl phosphine, approved by FDA for the treatment of rheumatoid arthritis, and it also showed therapeutic effect against parasitic, bacterial, and viral infections. The mechanism involved in the antimicrobial effects is based on the inhibition of redox enzymes, which induces cellular oxidative stress and intrinsic apoptosis. Moreover, this drug leads to an anti-inflammatory effect, reducing cytokines production and stimulating cell-mediated immunity. Rothan et al. demonstrated the ability of Auranofin to inhibit the SARS-CoV-2 replication in human Huh7 cells at low micromolar concentration and to reduce the expression of cytokines induced by virus infection, highlighting the potential of Auranofin to contrast with SARS-CoV-2 infection. Promising results regarding the employment of Auranofin as an antiviral drug were also obtained by Gil-Moles et al. [97]. The authors demonstrated that Auranofin and some other gold metallo-drugs (organometallics containing either a N-heterocyclic carbene or an alkynyl ligand) can interact not only with the SARS-CoV-2 spike protein, but also with the papain like protease (PLpro), a cysteine protease that may be a target for gold-based drugs due to their ability to interact with sulfur-containing molecules. The presence of exchangeable chloride and phosphane ligands is fundamental for a stronger interaction of the Au center with the enzyme. The activity of the tested gold complexes against PLpro is correlated with their ability to remove Zn${}^{2+}$ ions from the labile zinc center of the enzyme.

**Figure 5:**
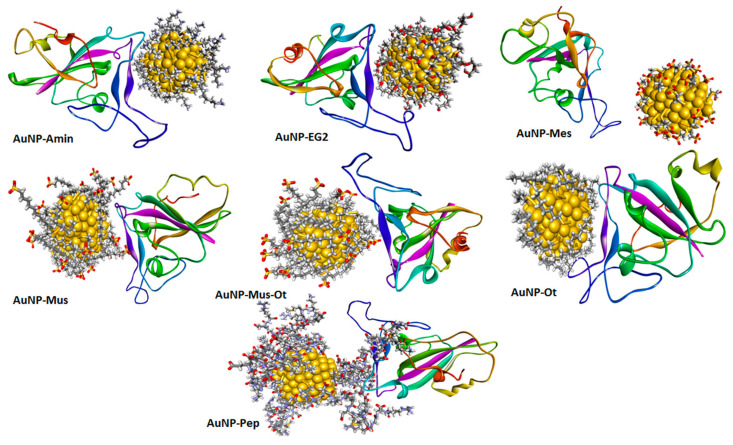
Simulation outcomes concerning the structures of some RBD complexes with Au NPs. Amin: 8-mercaptooctan-1-aminium; EG2: 2-(2-(6-mercaptohexyl)oxy)ethoxy)ethan-1-ol; Mes: 3-mercaptoethylsulfonate; Mus: Undecanesulfonic acid; Mus-Ot: Undecanesulfonic acid/Octanethiol; Ot: Octanethiol; Pep: Cys-Gln-Thr-Asp-Lys-His-Glu-Glu-Asp-Tyr-Gln-Met-Lys-Gly-Asp-Arg. Reprinted with permission from A. Mehranfar and M. Izadyar. Theoretical Design of Functionalized Gold Nanoparticles as Antiviral Agents against Severe Acute Respiratory Syndrome Coronavirus 2 (SARS-CoV-2). *Journal of Physical Chemistry*, Letters 2020, 11, 24, 10284–10289 [98]. Copyright 2020 American Chemical Society.

Cirri et al. also found that the organo–gold (III) compound Aubipyc possesses antiviral properties [87]. Through a computational study, the antiviral action of Aubipyc appeared due to the metalation of suitable metal-coordinating sites on viral proteins; that is, the deprotonated forms of cysteine and selenocysteine, evidencing the main role played by the pH of the milieu in determining the occurrence of metalation. However, Aubipyc showed a low selectivity index, which makes it not very suitable for in vivo tests. In the experiments by Cirri and coworkers, Auranofin showed no antiviral effect, maybe for the different experimental approach used in comparison with those performed by Rothan et al. [96]. Using molecular dynamics simulations, Mehranfar and Izadyar [98] investigated the anti SARS-CoV-2 ability of Au NPs functionalized by a new peptide (AuNP-Pep) based on the 15 ACE2 amino acids, which have considerable interaction with RBD (Figure 5). The findings showed that AuNP-Pep has a high ability to establish strong interactions with the S protein RBD of SARS-CoV-2, covering the whole binding surface of RBD using the peptide groups.

#### 3.1.3. Copper-Based Nanomaterials

Cu was the first metal to be declared an effective metallic antimicrobial agent in 2008 by the U.S. Environmental Protection Agency (EPA). Unlike bacteria, viruses do not develop mechanisms of resistance to copper ions, leading to their high susceptibility towards this metal and its derivatives [99]. Antiviral activity of copper compounds seems to depend on the release of Cu${}^{2+}$ ions in solution, which generates ROS, leading to the loss of genome integrity, lipid peroxidation, and deactivation of viral enzymes. A contact mechanism, based on metal ion binding and especially effective on the enveloped virus, has been described [100].

**Figure 6:**
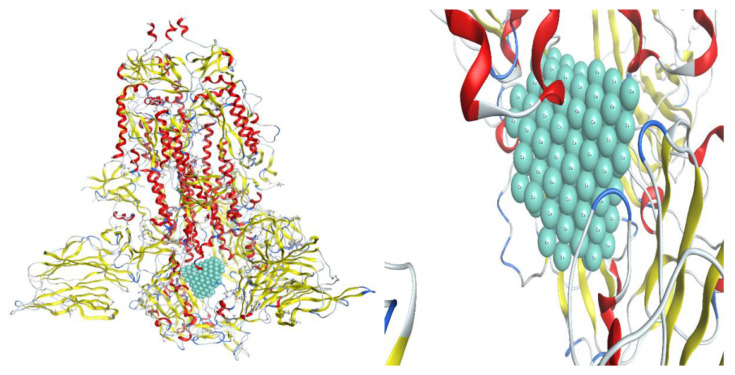
Scheme of the molecular docking involving the conical Cu NPs and the target spike glycoprotein (PDB ID: 6ZGG), together with the pharmacophore. Reprinted from *Journal of Molecular Structure*, Vol 1253, Aallaei et al. Investigation of Cu metal nanoparticles with different morphologies to inhibit SARS-CoV-2 main protease and spike glycoprotein using Molecular Docking and Dynamics Simulation, Art. N. 132301, Copyright (2022) [102], with permission from Elsevier.

However, other mechanisms leading to an antiviral effect cannot be excluded. For instance, CuO NPs can interact with Hepatitis C virus glycoproteins [101] and suppress its infectivity in the replication stage by inhibiting the activity of one of the required transcription factors [18]. Refat et al. [30] studied, via molecular docking analysis, the interaction of SARS-CoV-2 protease with Cu(II) complexes of deoxycholic acid (used in various fields of human medicine and food industry), evidencing their ability to interact with residues of this target protein. A computational study by Aallaei et al. [102] demonstrated the correlation between the shape of Cu NPs (spherical, conical, and cylindrical) and their interaction with SARS-CoV-2 main protease and spike glycoprotein (Figure 6). Cylindrical and conical Cu NPs were more efficient than spherical ones. The findings suggest a possible employment of Cu NPs as disinfectant.

SARS-CoV-2 inactivation may be also induced by treatment with copper iodide (CuI) NPs [103]. The virucidal action of CuI NPs is related to their ability to destruct viral S and N proteins, although viral genome damage caused by CuI treatment was also observed. This virucidal action may be mediated through direct and indirect mechanisms related to cuprous ions (Cu${}^{+}$) release and ROS production. In fact, Cu${}^{+}$ generates hydroxyl radicals both in the presence of H${}_{2}$O${}_{2}$, via a Fenton-like reaction, and in the absence of H${}_{2}$O${}_{2}$ [104]. The virucidal activity was also maintained using CuI-doped film and fabric. So, CuI NPs could be applied to produce coatings for high-touch surfaces, masks, protective clothing and, due to their good biocompatibility, could also be employed in hand hygiene products.

Jung et al. [105] showed the excellent antiviral performance of a copper-coated polypropylene (PP) filter face mask prepared by depositing a copper thin film (20 nm) on a spunbond PP filter surrounding a KF94 face mask. Oxygen ion beam pretreatment was employed to improve the film adhesion on the PP fibers and avoid copper film detachment (which should be a significant hazard for the possible inhalation of film particles) resulting in cuprous and cupric oxide formation.

Clay-based materials may be used for different biomedical purposes due to their interesting properties, which include large surface areas and adsorption capacity, thermal and chemical stability, good biocompatibility, and low cost [106]. Materials based on kaolin [Al${}_{2}$Si${}_{2}$O${}_{5}$(OH)${}_{4}$], a 1:1 clay mineral, can retain the virus in their surface, damaging viral proteins and thus blocking cell virus penetration [107]. Their antiviral properties may be improved, including metal NPs in the clay matrix, in this way: Rius-Rocabert et al. developed a nanohybrid system consisting of Ag or CuO NPs supported on kaolin plates [108]. Both materials showed a strong reduction in viral infectivity against SARS-CoV-2, as well as other enveloped and non-enveloped viruses, with a mechanism related to virus adsorption on kaolin plates and virucidal activity of the released metallic NPs and ions. Another advantage of this nanocomposite, to be used as a disinfecting antiviral, is that kaolinite plates act as a dispenser for the metallic NPs, avoiding a discharge in environment, and so limiting their environmental toxicity.

Almalki et al. have synthetized a new thiazole derivative to inhibit COVID-19 using Co(II) and Cu(II) complexes [109]. The results of their spectral analysis have been confirmed by theoretical calculations, explaining the details of the interactions with two COVID-19 proteins (namely the structures 6lu7 and 7bz5 from Protein Data Bank), comparing the antiviral properties of this new thiazole derivative with those of arbidol, avigan, and idoxuridine, three compounds already used in in the treatment of COVID-19. Their outcomes indicate that only the new thiazole derivative and idoxuridine possess antiviral properties contrary to those of arbidol and avigan, although these last ones continue to be used to defeat COVID-19.

#### 3.1.4. Zinc-Based Nanomaterials

Zn is an essential metal and plays different roles in cell metabolism (including proliferation and differentiation) and in immune response [110]. The role of Zn is related to its function as a cofactor in enzymes and binding several proteins; it can modulate their structural and/or regulatory functions [111]. Zn is also known to possess not only virucidal but also antiviral properties affecting virus replicative cycle [112,113,114].

**Figure 7:**
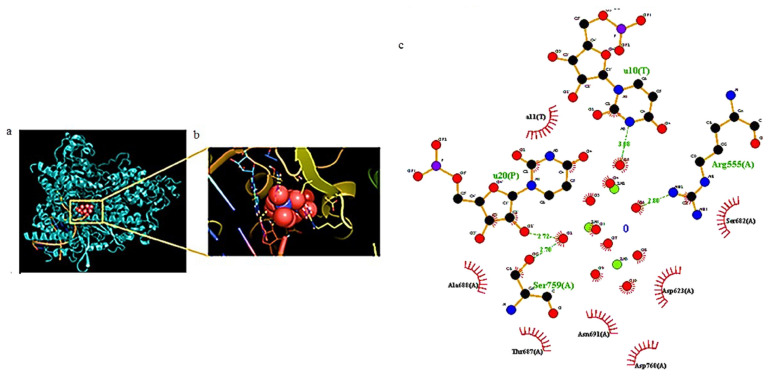
(**a**) Docking results of ZnO NPs interacting with amino acids of COVID-19 RdRp and (**b**) corresponding zoom. (**c**) The related ligand–protein interaction diagrams. The dashed green lines indicate hydrogen bonds. Figure reprinted from Ref. [121] under the terms of the Attribution-NonCommercial-NoDerivatives 4.0 International (CC BY-NC-ND 4.0) license.

Several authors have studied the effectiveness of Zn${}^{2+}$ ions against SARS-CoV-2 and the involved mechanisms due to the urgent need to identify efficient treatments for COVID-19 [115,116]. Pormohammad et al. [117] used molecular modeling to demonstrate that Zn can bind COVID-19 RdRp and 3CLpro. This bond influences the folded conformation and/or activity of these viral proteins, modulating viral replication. It was demonstrated that Zn${}^{2+}$ ions are able to bind the catalytic dyad of SARS-CoV-2 main protease (Mpro) via metal coordination bonds with His41 and Cys145 residues [118]. However, when studied by Grifagni et al. [119] using X-ray crystallography, the high affinity of the protein for Zn${}^{2+}$ ions appeared to be insufficient to produce its inactivation. Tao et al. demonstrated the ability of Zn gluconate (a common zinc supplement) to inhibit the proteolytic activity of PLpro and Mpro [120]. Crystallographic studies revealed two potential Zn${}^{2+}$-binding sites, one in the dyad catalytic center, and the other located on the surface of the protein structure. Furthermore, intracellular Zn${}^{2+}$ level can increase by combining Zn gluconate with hinokitiol (a small molecule ionophore), with an additional potential benefit in the employment of Zn supplements for COVID-19 treatment. A lower affinity towards Mpro compared to other SARS-CoV-2 targets, including the ACE2 receptor and SARS-CoV-2 RdRp, was also confirmed, studying the interaction of these proteins with ZnO via in silico docking studies (Figure 7). The binding affinity of ZnO NPs appeared ranked in the following descending order: ACE2 > SARS-CoV-2 RdRp > SARS-CoV-2 Mpro [121].

**Figure 8:**
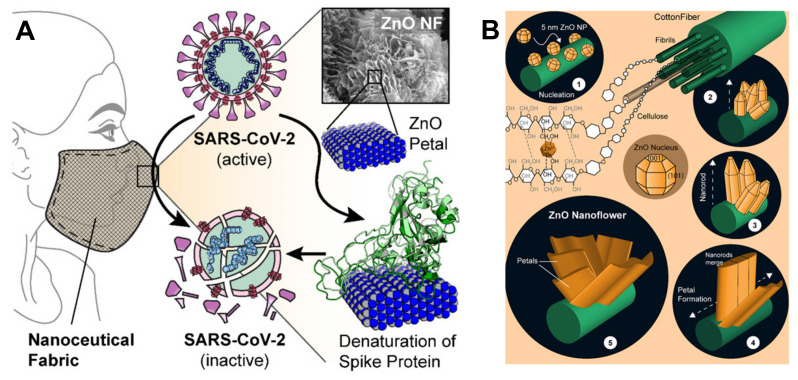
(**A**) Schematic representation showing how ZnO petals added to nanoceutical cotton fabric may induce a denaturation of the spike protein; (**B**) The five steps for growing ZnO nanoflowers on cotton cellulose fibers. Reprinted with permission from Adhikari et al. Nanoceutical Fabric Prevents COVID-19 Spread through Expelled Respiratory Droplets: A Combined Computational, Spectroscopic, and Antimicrobial Study. *ACS Applied Bio Materials*, 2021 4 (7), 5471–5484. Copyright 2021 American Chemical Society [122].

Adhikari et al. proved that the favorable interactions between SARS-CoV-2 spike protein, spherical ZnO NPs, and different facets of the ZnO nanostructure induce a denaturation of spike proteins (Figure 8A), which may lose their pathological function consisting of binding the ACE2 receptors in human lung epithelial cells [122]. The non-toxic Zn nanomaterial was duly added to nanoceutical cotton fabric to produce a membrane filter to employ in face mask fabrication (Figure 8B); this material revealed excellent bactericidal efficiency against *Pseudomonas aeruginosa*, used to mimic the coronavirus.

Sportelli et al. verified the anti-SARS-CoV-2 activity of ZnO NPs ecofriendly produced in the presence of both cationic and anionic stabilizers (cetyltrimethylammonium bromide—CTAB, poly-diallyl-(dimethylammonium) chloride—PDDA, poly (sodium 4-styrenesulfonate)—PSS). When tested in vitro, the PDDA-ZnO NPs induced a decrease in viral load between 70% and 90%; however no contribution was given by PDDA itself to the antiviral activity of the system. Then, PDDA-ZnO NPs were embedded into polyethylene oxide (a biodegradable and non-toxic polymer) film to reach a coating for frequently touched surfaces, with promising results, despite the polymeric material limiting the active surface of ZnO NPs to exert ionic release and subsequent antiviral activity [123].

A nano-spray disinfectant against SARS-CoV-2 based on ZnO NPs (with a size of about 50 nm) was designed by El-Megharbel et al. [124]. ZnO NPs showed anti-SARS-CoV-2 activity with potent antiviral activity at low concentrations (IC${}_{50}$ = 526 ng/mL), but with some cytotoxic effect to the cell host (cellular cytotoxicity [CC${}_{50}$] = 292.2 ng/mL), which limits its employment. To overcome the cytotoxicity issue and to reach high-performing anti-SARS-CoV-2 systems, the functionalization of ZnO NPs with biocompatible materials seems to be an interesting strategy. For example, ZnO NPs modified with polyethylene glycol, active against H1N1 influenza virus, showed lower cytotoxicity at highest concentrations [19].

Zn${}^{2+}$ ions embedded in polyamide fibers are a promising hybrid material in the fabrication of personal protective equipment thanks to their ability to decrease the SARS-CoV-2 titer by approximately 100-fold [115]. Two porous coating materials, based on submicrometer ZnO particles bound with silica menisci and ZnO tetrapods bound with polyurethane, were projected by Hosseini et al. as coating materials; the findings showed that infectivity is strongly related to material porosity and ability to absorb aqueous droplets [116].

The antiviral effect of Zn was also investigated as component of metal (II) aryl carboxylate complexes. Öztürkkan et al. synthetized Zn(II) 2-chlorobenzoate with 3-cyanopyridine (CNP), whose activity against Spike, Mpro, NSP12, NSP15, and NSP16 and ACE2 was studied via molecular docking [125]. However, these complex ligands—rather than the central metal atoms—are responsible for the interaction with target proteins.

#### 3.1.5. Nanomaterials Based on Other Transition Metals and Complex Metallic Alloys

The intrinsic antiviral activity of iron oxide nanoparticles (Fe${}_{m}$O${}_{n}$ NPs) is less investigated compared to that of the metal NPs previously discussed. Kumar et al. showed the antiviral activity of Fe${}_{m}$O${}_{n}$ NPs against the influenza virus HIN, supposing that this effect involves an interaction with the sulfur-bearing groups of viral hemagglutinin [126]. Molecular docking studies evidenced that Fe${}_{m}$O${}_{n}$ NPs induce variations in the conformation of the SARS-CoV-2 virus protein with the corresponding inactivation [127]. Furthermore, the bond between Fe${}_{m}$O${}_{n}$ NPs and the S1-RBD might induce ROS generation in turn, causing damage to the virus. To clarify its potential employment against different viral diseases, an iron-based system was also applied for the treatment of hepatitis C virus. The authors observed that the interaction depends on the chemical composition of Fe${}_{m}$O${}_{n}$ NPs: Fe${}_{3}$O${}_{4}$ NPs formed a more stable complex with S1-RBD, whereas Fe${}_{2}$O${}_{3}$ favored HCV E1 and E2.

Dinitrosyl iron complexes (DNICs) are constituted of Fe(NO)${}_{2}$ units (DNIUs) with two additional donor ligands, such as anionic thiolates or neutral N-donors. Synthetic DNICs have been investigated as NO donors for biomedical applications. Pectol et al. synthetized three dimeric thiolated DNICs as potential anti-COVID-19 agents. Computational studies have shown the capability of these DNICs to inhibit the SARS-CoV-2 Mpro through coordination of the monomeric DNIU to Cys145 [128].

Schiff bases derived from the condensation of an amino and a carbonyl compound form an important class of ligands that have the propensity to bind almost all metal ions via azomethine nitrogen. Recently, Mohamed et al. synthesized a new tridentate Schiff base ligand, 4-((1-(5-acetyl-2,4-dihydroxyphenyl)ethylidene)amino)-1,5-dimethyl-2-phenyl-1H-pyrazol-3(2H)-one, forming mononuclear chelates with Cr(III), Mn(II), Fe(III), Ni(II), Cu(II), Zn(II), and Cd(II) (metal/ligand ratio 1: 1); the binding affinity of the synthesized compounds towards the SARS-CoV-2 Mpro was examined via a molecular docking study [129]. Results indicate that the complex Cr(III) could possess good antiviral activity (better than that of the other metallic complexes), binding via H-donor with the amino acid residues of glutamic acid (Glu 166) and methionine (Met 49). The virucidal activity of titanium oxide nanoparticles (TiO${}_{2}$ NPs) is essentially correlated with the ability to generate ROS under UV irradiation, thus causing oxidative damages to biomolecules. The same ability was also shown by other semiconductor materials, such as Fe${}_{m}$O${}_{n}$ and ZnO, pointing out the high potential for virus inactivation and the potential use for production of textiles and objects with self-disinfecting properties [130]. Moreover, TiO${}_{2}$ NPs possess direct antiviral activity and may also have a role in viral clearance by stimulating immune mechanisms.

Recently, a new sterilization strategy using TiO${}_{2}$ nanotubes was proposed by Hamza et al. [131]. These TiO${}_{2}$ nanotubes have a powerful antiviral activity against SARS-CoV-2, very likely due to their ability to block viruses and inhibit their attachment to host cells. However, since a non-significant selectivity index (CC${}_{50}$/IC${}_{50}$ < 1) was calculated, the authors suggest their use as disinfectant due to the cytotoxicity on host cells. Unal et al. studied the antiviral and immunomodulatory properties of a series of transition metal carbides/nitrides (MXenes). MXenes have the general formula: M${}_{n+1}$X${}_{n}$T${}_{x}$, where M is an early transition metal (Ti, Nb, V, etc.), X is C and/or N, n is 1–4, and T${}_{x}$ represents the surface terminations (typically, O, OH, F, and Cl) [132]. The authors demonstrated that Ti${}_{3}$C${}_{2}$T${}_{x}$ exerts its viral inhibition activity against SARS-CoV-2 interacting with different protein targets, not only interfering with virus penetration but also with different steps of the virus life cycle. However, the antiviral property of this material is very likely not given by the presence of titanium, but is related to the polar negatively charged, and redox active surfaces of MXenes, which can strongly interact with viral proteins. In addition, this material has anti-inflammatory properties at the level of peripheral blood mononuclear cells.

**Figure 9:**
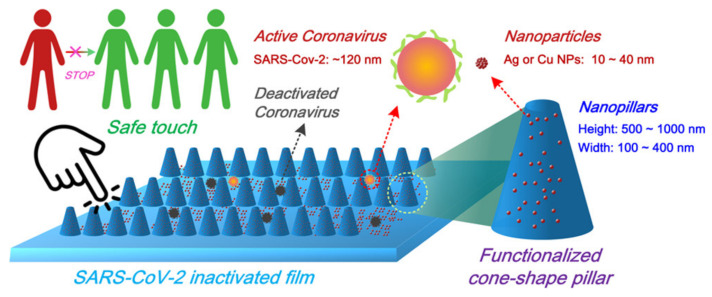
Simplified scheme about the design of functionalized nanoscale conical pillars with antiviral properties. Figure reused from Ref. [134] under the terms of CC BY 4.0 license.

The use of metal alloys and metal complexes in devices or as surface coating agents is turning out to be a promising approach. Cu alloys are known to be highly efficient antimicrobial systems; thus, Pan et al. prepared a copper–zinc nanowire (CuZnNW) ink to be sprayed on high-touch surfaces [133]. CuNW inactivates SARS-CoV-2 faster with respect to bulk Cu, and the addition of a lower amount of Zn (0.16 at % zinc as measured by ICP-MS in the filtered CuZnNW ink) leads to an improvement in the virucidal activity. Zn stabilizes the copper ions release, and thus, the coating is effective for a longer time. Moreover, Zhou et al. [134] have developed novel plastic films finalized to increase the effective contact area between virus particles and the surface coatings and deactivate SARS-CoV-2. The system is based on Ag NPs and Cu NPs (the size of which vary between 10 and 40 nm), combined with nanoscale conical pillars, together with the addition of sodium dodecyl sulfate and polyvinyl acetate to ameliorate the features of the coating deposited on polyethylene terephthalate and polyethylene films (Figure 9).

Bello-Lopez et al. demonstrated the efficacy against SARS-CoV-2 spread of nanometric layers of bimetallic AgCu deposited on polypropylene fibers, to prepare reusable cloth masks. The virucidal performance is increased by the employment of bimetallic particles instead of single materials. Quantum chemistry calculations, employed to investigate the mechanism involved in the biocide contact action, confirmed that the addition of Ag-Cu NPs makes the polymeric fiber a better electron acceptor, producing damage to viral phospholipids and genetic material [135]. Similarly, Mosselhy et al. [136] showed that two Cu-Ag nanohybrids with higher amounts of Cu and lower amounts of Ag (around 65 wt% and 78 wt% and 7 wt% and 9 wt%, respectively) inhibited SARS-CoV-2 efficiently when used as surface coatings. Chang et al. formulated a metal nanocomposite containing spherical Au, Ag, and ZnO NPs (1 ppm, 5 ppm, and 60 ppm, respectively) and ClO${}_{2}$ (42.5 ppm) in aqueous solution. This material was able to inhibit six major clades of SARS-CoV-2 by preventing the binding of viral spike proteins to ACE2 receptor, and interfering with the syncytium formation [137]. The amounts of Au (<0.01 ppm), Ag (<0.05 ppm) and ZnO (<0.6 ppm) in the calculated 50% effective dose were in the range of concentrations approved to be used as food additives, while the amount of ClO${}_{2}$ (<0.425 ppm) is within the safety concentration range for drinking water. Furthermore, this material was also shown to efficiently inhibit human (H1N1) and avian (H5N1) influenza viruses, so that it may be used for prophylactic effect against SARS-CoV-2, but also against opportunistic infections frequently observed in COVID-19 patients. Given efficacy and safety findings, this material may be used by oral gargling, nasal spray, or nebulized inhalation. Moreover, Chuong et al. have reported direct virucidal activity against SARS-CoV-2 by two pentamethylcyclopentadienyl (Cp*) rhodium piano stool complexes, which may be used in antiviral therapy or as antiviral surface coatings [138]. Their outcomes showed that these complexes have low toxicity and their physical properties (e.g., hydrophobicity), and indeed their biological activity, can be tuned by changing the ligands’ structure.

### 3.2. Chitosan

Chitosan (CT) is a linear polysaccharide constituted of β-(1 ← 4)-linked d-glucosamine and N-acetyl-d-glucosamine, with a molecular weight range of 3800–200,000 Daltons. Commercial CT is obtained via alkaline treatment and deacetylation of chitin, the second most abundant polysaccharide present in shrimp shells, fungi etc. [139]. The easy and cheap route to synthetize CT, as well as its biodegradability, non-toxicity and low allergenicity, highlight the potentiality of this natural polymer for biomedical applications. CT showed significant activities against important fungal human pathogens and several pathogenic bacterial strains. Additionally, CT derivatives are characterized by a wide range of actions against bacteriophage, plant, and animal viruses [140].

In addition to its employment as vaccine adjuvant [141], in the last two years, the attention of the scientific community has focused on its use as an antiviral agent, a component of DDSs for antiviral agents, and material for protective fabrics to contrast SARS-CoV-2 diffusion [139,142,143]. CT was demonstrated by Alitongbieke et al. [144] to strongly bind the ACE2 and spike-RBD under normal physiological conditions. Furthermore, in vitro analysis indicated downregulation of ACE2 levels by CT in Vero E6 cells, decreasing the probability of spike-RBD binding to ACE2 and an ability of CT to exert an anti-inflammatory effect. When intranasally administered in hACE2 mice, CT prevents lung damage following virus binding with ACE2 protein. The findings also showed a downregulation of the ACE2 expression, very likely through activation of ADAM17, a metalloproteinase which improves the cleavage of the ACE2 extracellular juxtamembrane region (producing an effect apparently similar to protein downregulation). In accordance with these data, Kalathiya et al. [145] demonstrated the binding affinity of CT to an homotrimer pocket and the RBD of the S protein through molecular dynamics study. The antiviral activity of CT is affected by several factors, such as the natural source from which the chitin was extracted, the synthetic route adopted, the molecular weight, the deacetylation degree, the surface charge, and the chemical modifications [140]. The semi-crystalline structure of CT, due to the formation of hydrogen bonds among hydroxyl (OH) and amino (NH${}_{2}$) functional groups, limits the accessibility to these reactive groups on the polymer backbone, restricting CT biomedical activity. Indeed, the degraded CT shows higher antiviral activity compared with the starting one, even if it is not strong enough for use in clinical treatment, as shown against the Newcastle disease virus [146]. Therefore, deacetylation, depolymerization, and chemical modification and/or addition of reinforcing agents are useful strategies to improve the antiviral activity of CT [147]. It is demonstrated that the amino groups, particularly the cationic ones, play a key role in biological activity of CT. He et al. prepared 6-amine chitosan derivatives, enriched with bromine ions, which showed significant antiviral activity against Newcastle virus, based on inhibition of virus transcription as well as stimulation of immune responses [148]. Sulfated polysaccharides are also able to prevent the binding of the virus on the host cells by interacting with the glycoproteins present on the cell surface. In particular, sulphated-CT blocks the interactions between HIV-1gp120 and CD4 cell surface receptors [149]. Gao et al. [21] reported that 3,6-O sulphated chitosan, with sulfate content of 45.8%, inhibits the cell fusion, and thus, the entry inside cells of human papillomaviruses (HPVs), by directly binding to virus capsid protein or interfering with the PI3K/Akt/mTOR cell pathway to inhibit the host autophagic process required for virus cell penetration.

**Figure 10:**
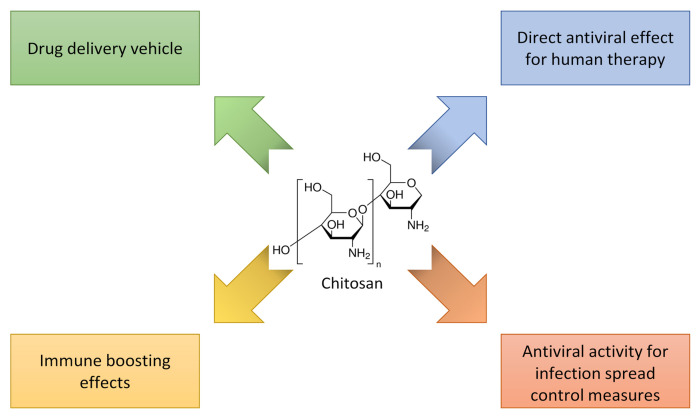
Possible applications of chitosan in COVID-19.

Considering this interesting performance of CT and its derivatives as antiviral agents, the attention is focused on them to fight SARS-CoV-2 infection, and the possible applications of chitosan to treat COVID-19 are summarized in Figure 10. Positively charged CT derivatives can inhibit the cell penetration of several members of the Coronaviridae family, thanks to the high amount of quaternary ammonium groups, which electrostatically bind the virus S proteins [150]. In fact, the cationic modified chitosan, N-(2- hydroxypropyl)-3-trimethylammonium chitosan chloride (HTCC), is a potent inhibitor of the coronavirus HCoV-NL63, hindering the viral interaction with its receptor, and thus its cell entry. HTCC also showed considerable blocking activity towards SARS-CoV-2 and MERS-CoV, probably due to the high molecular weight (50–190 kDa) and the high degree of quaternary ammonium functional groups (degree of substitution 57–77%) [151]. It is also effective against HCoV-NL63 virus, one of the most important causes of croup in children [152].

However, Pyrć et al. also demonstrated that a different quaternary ammonium CT with a low molecular weight (10–30 kDa) and low content of quaternary ammonium groups (<40 mol%) (N-palmitoyl-N-monomethyl-N,N-dimethyl-N,N,N-trimethyl-6-O-glycolchitosan, GCPQ) can hinder SARS-CoV-2 entry into cells [153]. Oligochitosans without the quaternary ammonium group were inactive in inhibiting coronavirus entry into cells [152], outlining the evident key role of quaternary ammonium to determine the antiviral action. GCPQ electrostatic binding to the virus was suggested as the most likely mechanism of viral inhibition to prevent viral entry into the host cells. CT contains many amino groups protonated at acidic pH, and this cationic charge enables CT-based systems to adhere and penetrate the mucosa of lung epithelial cells, facilitating drug cell internalization and accumulation. Furthermore, the mucoadhesive property improves the intercellular tight junctions at the level of lung epithelium. On the basis of the results obtained in vivo in a mouse model, GCPQ may be proposed for a possible employment as nasal spray in the prophylaxis of SARS-CoV-2 infection. Vörös-Horváth et al. [154] projected CT hydrogels with dicarboxylic acids (malic and glutaric acid) and loaded with three different ACE2 inhibitors (glycyrrhizic acid, baicalin, and emodin), finalized (due to their ACE2 inhibitor content and mucoadhesive property) to be used as nasal formulation to prevent SARS-CoV 2 infection. Hanafy and El-Kemary [155] developed a mucoinhalable delivery system composed of the polyphenols silymarin and curcumin (both of which recently demonstrated the ability to directly interact with SARS-CoV-2 proteins, and so prevent virus penetration and replication) encapsulated in NPs made of bovine serum albumin (BSA; characterized by non-toxicity, good stability, and high ability to encapsulate hydrophobic and hydrophilic drugs) and coated with CT as a mucoadhesive polymer. The system showed good antiviral in vitro efficacy against SARS-CoV-2 and a significant anti-inflammatory effect in vivo in oleic-acid-treated mice (a model of acute lung inflammation).

CT may be employed to ameliorate the properties of lung-targeted DDSs independent of its mucoadhesive properties. Ivermectin (IVM) exhibits antiviral activity against SARS-CoV-2, even if the repurposing of IVM for the treatment of COVID-19 presented challenges, primarily due to its low oral bioavailability. Zheng et al. [156] projected a red blood cell (RBC)-hitchhiking strategy for lung targeted delivery of IVM. They prepared IVM-loaded NPs based on poly(lactic-co-glycolic acid) (PLGA) and then coated with CT and the obtained NPs (mentioned as IVM-PNPs and IVM-CSPNPs, respectively non-coated and coated with CT) were adsorbed onto RBCs. This system appeared capable of enhancing IVM delivery to lungs, improve IVM accumulation in lung tissue, and inhibit the inflammatory response. Interestingly, RBC-hitchhiked cationic IVM-CSPNPs showed better properties than RBC-hitchhiked anionic IVM-PNPs. This is a recent hybrid delivery strategy in which NPs are adsorbed onto the surface of RBCs and subsequently scraped off the RBC surface due to the shear stress between RBCs and small capillaries. Then, the desorbed NPs were rapidly accumulated, mainly in the lung capillary after intravenous injection [157]. This technique enables parenteral delivery of insoluble drugs and, due to the long circulation property of RBCs, can greatly prolong the circulation and action time of drugs.

The addition of organic or inorganic materials to CT is currently employed to endow protective equipment with antiviral activity or to prepare antiviral disinfectants. Qingliang et al. prepared a CT based hydrogel, combining partially acrylated CT with polyvinylpyrrolidone, gelatin, fumed silica, coconut oil diethanolamide, fatty alcohol polyoxyethylene ether sodium sulfate, and a photoinitiator, to be used in medical spray or as additive in gloves [158]. Zhang et al. fabricated face masks with antiviral properties by adding CT thermal-bonded non-woven fabric as an antiviral functional layer [159]. The authors obtained a fibrous network structure constituted of ethylene-propylene and CT via carding and hot air through bonding. The system showed significant antiviral activity against Enterovirus 71. The antiviral activity was probably due to the electrostatic interactions between the positive charged CT and the negative charged virus [159]. Antiviral filters based on CT fibers coating with copper were fabricated by Xianming et al. [160]. These filters are able to trap and kill both bacteria and virus, representing an interesting composite for the protection of several kinds of medical equipment. Favatela et al. have prepared hydrogel formulations by employing the citric acid and copper salts as enhancers of CT action [161]. The biopolymeric-based formulations, with different content of the constituted materials, showed suitable properties for the textile impregnation, as well as antiviral action.

### 3.3. Carbon- and Polymer-Based Nanomaterials

**Figure 11:**
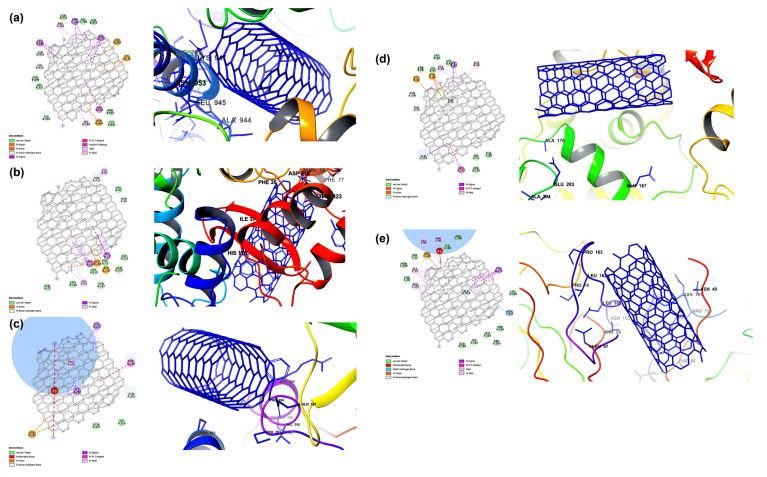
Molecular docking outcomes concerning the interaction potential of carbon nanotubes towards the following targets of SARS-CoV-2: (**a**) spike glycoprotein, (**b**) RdRp, (**c**) Mpro, (**d**) papain-like protease and (**e**) RNA-binding domain of nucleocapsid protein. Figure reprinted from Ref. [168] under the terms of the Creative Commons CC-BY-NC-ND license.

Carbon-based nanomaterials, thanks to their interesting properties, are deeply investigated for several therapeutic applications, including antiviral performances [162,163,164,165]. Their potentiality as biomedicine tools is mainly ascribed to their ability to cross the cellular membrane via different pathways and directly interact with several biomolecules, such as proteins [166,167]. Skariyachan et al. [168] investigated, via molecular docking and molecular dynamic simulation analysis, the possible interactions between carbon nanotubes/fullerene with the putative targets of SARS-CoV-2 (spike glycoprotein, RNA-dependent RNA polymerase, main protease, papain-like protease, and RNA binding domain of the nucleocapsid proteins) as shown in Figure 11.

**Figure 12:**
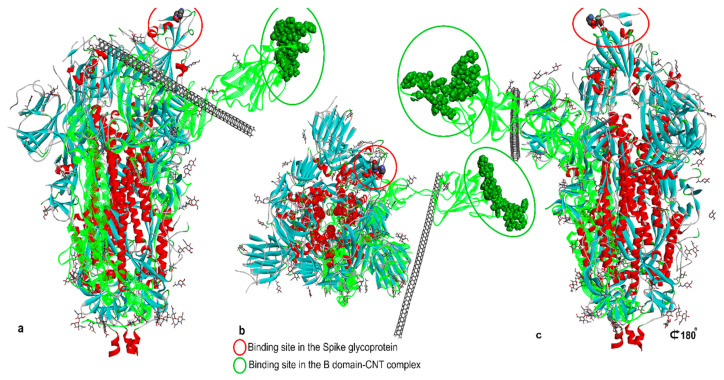
(**a**–**c**) MD simulation outcomes showing the evolution of the interactions between SWCNTs and SARS-CoV-2 spike glycoprotein. The green chain corresponds to the B domain of the spike glycoprotein simulated in the presence of SWCNTs, whereas the blue–red one is the spike glycoprotein (PDB ID: 6VYB). Reprinted from *Computers in Biology and Medicine*, Vol 136, Jomhori et al. Tracking the interaction between single-wall carbon nanotube and SARS-Cov-2 spike glycoprotein: A molecular dynamics simulations study, Art. N. 104692, Copyright (2021) [169], with permission from Elsevier.

Both carbon nanotubes and fullerene showed stable and potential interactions with all these targets, with carbon nanotubes showing a better performance. Moreover, the binding affinity of carbon nanotubes and fullerene towards protein targets was significantly higher with respect to those calculated for several drugs and other materials suggested to be effective against SARS-CoV-2. Although the absorption, distribution, metabolism, and excretion (ADME) predictions of the carbon nanomaterials were within an acceptable range, the calculated drug-likeness molecular descriptors, as well as toxicity predictions, were unfavorable. Similar data were obtained by Jomhori et al. [169], who studied the interactions between single-walled carbon nanotubes (SWCNTs) and SARS-CoV-2 spike glycoprotein via molecular dynamics simulation. The authors demonstrated that the binding of SWCNTs with B monomer of the spike glycoprotein induces an alteration in the tertiary structure of the B domain (Figure 12). All this could probably inhibit the interactions between the ACE2 receptor and the SARS-CoV-2 S protein, blocking the virus’ entry into the cells.

Suárez et al. [170] studied the antiviral activity of a steroid–endohedral fullerene (H${}_{2}$@C${}_{60}$) hybrid system, obtained via covalent conjugation of H${}_{2}$@C${}_{60}$ with dehydroepiandrosterone (DHEA). DHEA has been recently proposed as a drug used to mitigate the pro-inflammatory symptoms of COVID-19 [171]. A molecular docking simulation of the binding with PLpro and 3CLpro evidenced that the steroid–H${}_{2}$@C${}_{60}$ hybrid system strongly blocks the active site area of both proteases through hydrophobic interactions and hydrogen bonding. Further investigations revealed that the hydrophobic interactions established by H${}_{2}$@C${}_{60}$ moiety with residues of the PLpro protein cannot play a key role in the enzyme inhibition process, so H${}_{2}$@C${}_{60}$ is not a good PLpro inhibitor; as for 3CLpro, H${}_{2}$@C${}_{60}$ is not able to form strong interactions through hydrogen bonds. These findings outline the importance of the functionalization with a steroid moiety to improve the action of H${}_{2}$@C${}_{60}$ against SARS-CoV-2.

**Figure 13:**
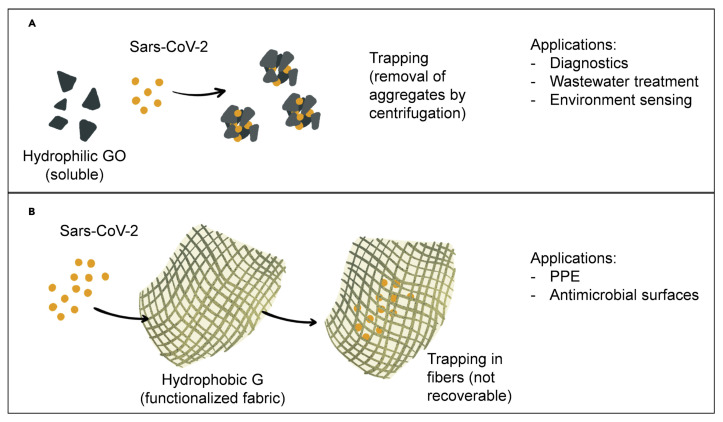
Schematic preparation and potentential applications of nanohybrid systems based on (**A**) hydrophilic GO and (**B**) hydrophobic graphene materials employed to defeat SARS-CoV-2. Figure reprinted from Ref. [173] under the terms of the Creative Commons CC-BY-NC-ND license.

Molecular docking studies showed a high ability of graphene oxide (GO) nanosized sheets to interact with S protein, ACE2, and the ACE2-bound spike complex, although GO binds more strongly to ACE2 and S protein than the ACE2-spike complex [172]. This is due to 12 hydrogen bonds, 2 hydrophobic, and 1 electrostatic interaction computed for ACE2, in comparison with the 7 hydrogen bonds and 2 hydrophobic with ACE2-bound spike complex. Moreover, the study revealed the capability of GO nanosheets to hinder virus infectivity, showing in vitro antiviral activity against three different clades of SARS-CoV-2. The antiviral activity of graphene (G) and GO was also confirmed by De Maio et al. by means of in vitro experiments on Vero cells infected with SARS-CoV-2. Furthermore, in this study, G and GO were used to functionalize polyurethane or cotton to obtain new hybrid materials (Figure 13), which appeared able to in vitro eradicate SARS-CoV-2 infectivity, and thus are potentially useful for production of personal protective equipment (PPE) [173]. Due to its hydrophilic properties, GO could also be employed for water treatment and air purification.

Since polylysines have been reported to possess an antiviral effect against RNA and DNA viruses, Stagi et al. [174] prepared NPs based on hyperbranched polylysine, synthesized by boric-acid-catalyzed thermal polymerization of L-lysine. The authors demonstrated that these NPs (200–300 nm) have good antiviral activity, acting at different stages of the SARS-CoV-2 life cycle. The positive surface charge of these hyperbranched polylysine NPs (which are only slightly larger than the virus nanoparticles), together with the branched structure of the polymer, should be responsible for a strong interaction with the virus, since generally, viral particles have a negative charge at neutral pH. In addition, polylysine NPs induced no antiviral effect.

Inorganic polyphosphate (polyP) is a polymer released from blood platelets, and is involved in blood coagulation [175]. Neufurth et al. [176] showed that polyP3 and polyP40 (3 and 40 Pi units), soluble or encapsulated in silica NPs (to avoid enzymatic hydrolysis of polyP by alkaline phosphatase), can efficiently inhibit the binding of SARS-CoV-2 S protein to ACE2. This occurs via electrostatic interaction of polyP with basic amino acids on the RBD target; this effect was observed at a concentration of 1 μg/mL, similar to the physiological level of polyP in the blood. Importantly, this material also maintains its properties if added to a mouth-flushing solution, so that it might be used to prevent the infection at the level of the oropharyngeal cavity. Finally, due to the physiological role of polyP, these findings appear very interesting, since severe coagulopathies and thrombocytopenia often occur in COVID-19 patients.

### 3.4. Cytomimetic Nanomaterials

As stated in the previous sections, the main approach to developing novel therapies against COVID-19 is focused on target cells and their protein receptors. This is particularly important, since during the last 2 years, an increasing number of mutated strains of SARS-CoV-2 have been identified. As consequence of these mutations, the S protein has a higher binding affinity for host ACE2, resulting in higher transmission of SARS-CoV-2, and also representing a serious problem for the effectiveness of therapies targeting the virus proteins. Recently, cytomimetic nanomaterials have been projected to mimic characteristics of human cells naturally targeted by SARS-CoV-2 and/or those that have a role in COVID-19. The main part of them is based on cell membranes, taking advantage of functional elements present on them. Other nanosystems are finalized to vehicle proteins functioning as SARS-CoV-2 receptors or induce their expression. These innovative nanotechnological approaches are often mentioned as “nanodecoys” or “nanosponges” or “nanocatchers” and offer numerous therapeutic opportunities due to their great potential for neutralizing SARS-CoV-2 infectivity, as well as for alleviating the cytokine storm caused by SARS-CoV-2 infection.

Zhang et al. [177] designed hACE2-containing nanocatchers (NCs) as the competitor with host cells for virus binding to protect cells from SARS-CoV-2 infection. The human ACE2 (hACE2)-containing NCs were produced from the cellular membrane of genetically engineering human embryonic kidney (HEK) 293T cells stably expressing ACE2 (hACE2-293T), obtained by 293T cells infected with lentivirus encoding hACE2. The neutralizing ability of these NCs was demonstrated using vesicular stomatitis virus (VSV)-based pseudotyped SARS-CoV-2, since the pseudovirus containing the S protein of coronaviruses is a reliable and safe tool for screening and characterizing new drugs with anticoronavirus infection activities [178]. These NCs exhibited excellent neutralization ability against pseudoviruses of both wild-type SARS-CoV-2 and the D614G variant. Since the lung is the most vulnerable organ for COVID-19, an inhalable formulation was produced by mixing hACE2-containing NCs hyaluronic acid (HA) and sucrose. The mucoadhesive excipient HA can significantly prolong the retention of NCs in the lung after inhalation, and the employment of the cryoprotectant sucrose during lyophilization increases the NCs stability during long-term storage without loss of their activity. This preparation, due to its mucoadhesive properties, possessed improved lung retention, as shown when intratracheally delivered into male Balb/c mice via inhalation from a microsprayer aerosolizer. Importantly, powerful antiviral activity was demonstrated in hACE2-expressing NSG mice receiving intratracheal administration of the NC preparation and challenged with pseudotyped SARS-CoV-2 after NC inhalation. Similarly, Wang et al. [179] prepared membrane NPs using HEK-239T cells highly expressing ACE2 (ACE2-NPs); these NPs showed a potent capacity to block SARS-CoV-2 infection. ACE2-NPs reduced S1 recruitment and cell penetration when tested on HK-2 human renal tubular epithelial cells; similar results were also obtained when the D614G mutation was employed. The pretreatment with ACE2-NPs reduced the penetration of SARS-CoV-2 S pseudovirions into HK-2 cells, (which abundantly express ACE2, TMPRSS2, and furin). The antiviral effectiveness of intravenously administered ACE2-NPs was also confirmed in vivo in hACE2-expressing C57 mice injected with S pseudovirions.

**Figure 14:**
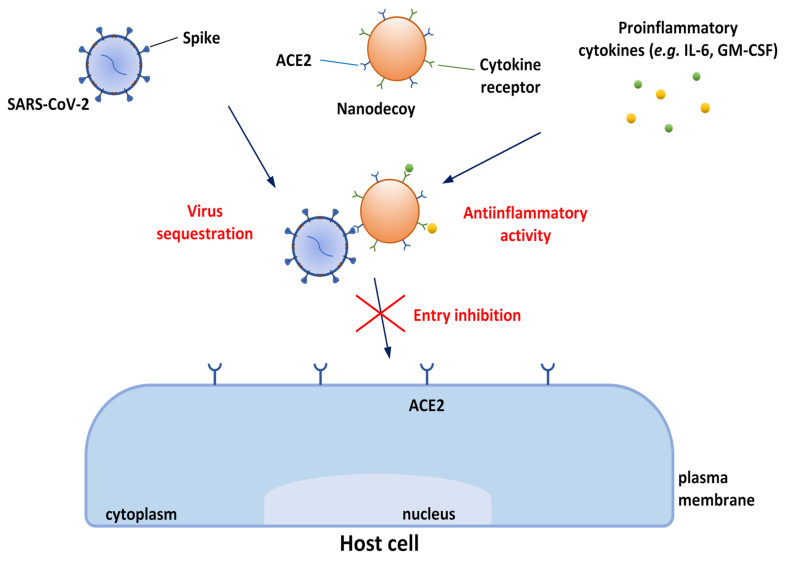
Cytomimetic nanosystems are innovative materials in the fight against COVID-19. The present figure is a schematic representation of the nanodecoy projected by Rao et al. [181].

Zhang et al. [180] created cellular nanosponges made of human-cell-derived membranes obtained from human lung epithelial type II cells or human macrophages (the cells which are naturally targeted by SARS-CoV-2) and coated onto PLGA NP cores. These nanosponges displayed the same receptors (ACE2 and CD147) used by the virus for penetration into the host cells. The neutralizing ability of these nanosponges, in comparison with nanosponges made from red blood cell membranes, was demonstrated using live SARS-CoV-2 viruses on Vero E6 cells. One has to mention that macrophage-derived nanosponges might have a role not only in the prevention of viral penetration through CD147 infection, but also in counteracting the hyperinflammation response to the virus, since macrophages play a significant role in the pathogenesis of infections such as those from SARS-CoV and MERS-CoV. Rao et al. [181] projected a nanodecoy against COVID-19, allowing both virus neutralization and cytokine neutralization (Figure 14). This is important because very few drugs are known to target the cytokine storm associated with the late stage of infection. In particular, mAbs targeting the proinflammatory cytokine IL-6 and the myelopoietic growth factor granulocyte-macrophage colony-stimulating factor (GM-CSF) have been investigated for their potential capability to counteract inflammation triggered by SARS-CoV-2 [182].

The nanodecoy was produced by fusing cellular membrane nanovesicles derived from engineered 293T/ACE2 cells and from human myeloid mononuclear THP-1 cells, so that the nanodecoys present both the receptor ACE2 for SARS-CoV-2 and the cytokine receptors CD130 and CD116, respectively, for IL-6 and GM-CSF, and thus can concurrently neutralize SARS-CoV-2 and inflammatory cytokines. These nanodecoys were shown to effectively protect host cells from the viral infection, as shown against pseudotyped SARS-CoV-2 on human hepatoma Huh-7 cells and against authentic SARS-CoV-2 on Vero-E6 cells. Due to the abundance of cytokine receptors on their surface, they efficiently neutralize inflammatory cytokines; in fact, when solutions containing IL-6 and GM-CSF were incubated with nanodecoys, the NPs appeared able to bind inflammatory cytokines and remove them from the supernatant. Consistent data were also obtained when nanodecoys were tested using THP-1 cells stimulated by lipopolysaccharide (LPS) to up-regulate inflammatory cytokines and mimic the infection-related inflammatory state. These results evidence the potential employment of nanodecoys in suppression of a cytokine storm. Finally, the in vivo performance of nanodecoys were tested in an acute lung inflammation (ALI) mouse model. To induce ALI, the mice were first intratracheally inhaled with LPS and then received intratracheal inhalation of nanodecoys. Nanodecoys suppressed lung injury and decreased the IL-6 and GM-CSF levels in the lung bronchoalveolar lavage fluid, in comparison with the findings obtained after LPS challenge only.

Li et al. [183] projected ACE2 nanodecoys derived from human lung spheroid cells (LSCs). LSCs developed by this research group are a natural mixture of resident lung epithelial cells (containing both type I and II pneumocytes) and mesenchymal cells. In particular, ACE2 is present on the membrane of AQP5+ type I pneumocytes and SFTPC+ type II pneumocytes, two subpopulations within LSCs. Since resident lung cells express ACE2 and more than 80% of ACE2-expressing cells in lung tissue are type II pneumocytes, these ACE2 nanodecoys derived from LSCs can bind and neutralize SARS-CoV-2. Nanodecoys were able to bind and neutralize spike S1 in vitro, and to bind SARS-CoV-2 mimics, fabricated by modifying a lentivirus without spike S1 to express spike S1 conjugated on its surface. In addition to demonstrating the lung retention and the biodistribution of LSC-nanodecoys in normal mice after inhalation, as well as the accelerated clearance of SARS-CoV-2 mimics from the lungs of mice receiving inhaled nanodecoys 24 h post viral exposure, the authors demonstrated the efficacy of this system, used by inhalation, in a pilot non-human primate study on six cynomolgus macaques (a model which reproduces many clinical symptoms of SARS-CoV-2 infection due to strong viral replication at pulmonary level) challenged with live SARS-CoV-2 via intranasal and intratracheal routes. Finally, Chen et al. [184] developed liposomal-based nanotraps capable of capturing SARS-CoV-2 and facilitating macrophage phagocytosis. The starting point is that macrophages engulf apoptotic cells by recognizing phosphatidylserine on the outer leaflet of the plasma membrane of apoptotic cells, while phosphatidylserine coatings enhance the uptake of liposomes by macrophages [185,186]. NPs prepared by Chen et al. were made by a solid polylactic acid (PLA) core acting as a ‘‘cytoskeleton’’ and a lipid shell based on 1,2-dioleoyl-sn-glycero-3-phospho-L-serine (DOPS) enveloping the PLA core. Their surfaces were functionalized with either recombinant ACE2 proteins or anti-SARS-CoV-2 neutralizing antibodies and a phagocytosis-inducing ligand (phosphatidylserine). The successful functionalization of these nanotraps (whose optimal composition was 500-nm core size and 15% surface phosphatidylserine) was demonstrated by in vitro experiments on differentiated THP-1 macrophages. Ultimately, these nanotraps appeared effective in the neutralizing assay against authentic SARS-CoV-2 on Vero E6 cells and blocked SARS-CoV-2 pseudovirus infection on HEK293T-ACE2 cells and on A549 epithelial cells. Being composed of FDA-approved polymers and lipids, these systems demonstrated an excellent safety profile not only in vitro, but also in vivo after intratracheal administration in WT B6 mice. More interestingly, these nanotraps inhibited pseudotyped SARS-CoV-2 infection in live human lungs in an ex vivo lung perfusion system. This model is more clinically relevant than those based on lung organoids, which cannot reproduce the whole-organ response to viruses, and less expensive than non-human primate models.

ACE2-functionalized biomaterial particles have been demonstrated to be useful as a prophylactic countermeasure against SARS-CoV-2 infection. Strong and Pelaez [187] projected innovative particles fabricated through the conjugation of recombinant human ACE2 (rhACE2) onto HisPur magnetic particles. In vitro experiments showed that this system is able to bind the RBD of recombinant SARS-CoV-2 spike protein and, when tested on primary human epithelial cell cultures and on primary ACE2 overexpressing HEK293 cells, to compete with endogenous cellular ACE2 targets. Positive results were also obtained in vivo following inhalation of rhACE2-cytomimetic particles by mice prior to their exposure to aerosolized spike protein [187].

It is well known to the researchers working in this field that hACE2 can be cleaved by peptidases at the neck region of the extracellular segment, releasing a soluble form of hACE2 (hsACE2) which is enzymatically active [188,189]. Since the RBD of SARS-CoV-2 binds to the extracellular domain of hACE2, hsACE2 protein can prevent viral infection through competitive inhibition. Taking this concept into account, Kim et al. [190] projected a mRNA-based nanotherapeutic producing the decoy hsACE2 protein to inhibit SARS-CoV-2 infection. To this end, a synthetic mRNA encoding for hsACE2 was produced; this mRNA was loaded into lipid nanoparticles (LNPs) capable of transfecting human cells, and so enhancing hsACE2 secretion. These LNPs were composed of ionizable lipid dilinoleylmethyl-4-dimethylaminobutyrate (DLin-MC3-DMA), helper lipids (1,2-distearoyl-sn-glycero-3-phosphocholine (DSPC) and cholesterol or β-sitosterol), and 1,2-myristoyl-sn-glycerolmethoxy poly(ethylene glycol) 2000 (DMG-PEG2k). When intravenously administered in mice LNPs led to hepatic delivery of the mRNA and secretion of hsACE2. The potential therapeutic efficacy of this nanosystem, administered through instillation into the lungs of female BALB/c mice, was demonstrated by detection of hsACE2 in the bronchoalveolar lavage fluid. This system represents an innovative example of gene therapy against COVID-19 and could be employed to block SARS-CoV-2 both in bloodstream and in lung.

### 3.5. Nanomaterials for Photothermal, Photodynamic, and Photocatalytic Treatment

**Figure 15:**
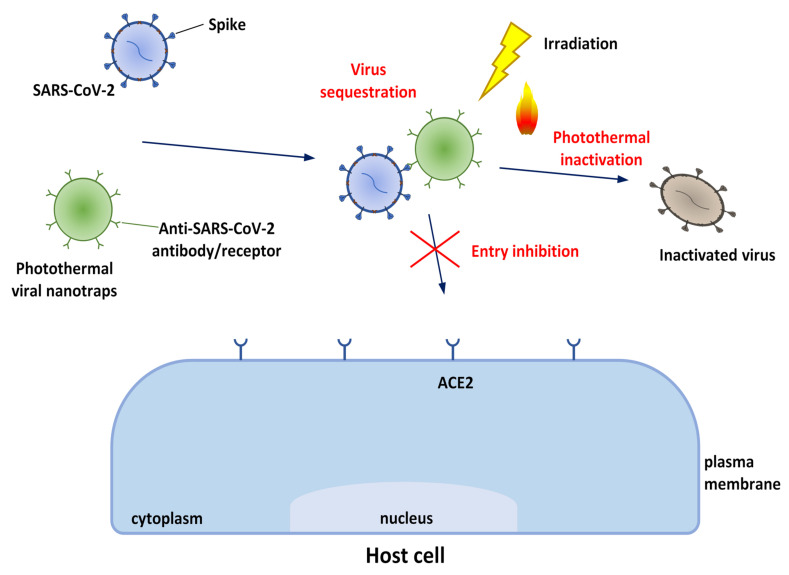
Schematic representation of nanomaterials for virus sequestration and photothermal inactivation.

High-affinity neutralizing mAbs against SARS-CoV-2 have the advantage of bypassing the risk of antibody-dependent enhancement, since they have an affinity for the SARS-CoV-2 S protein that is much higher than that for ACE2. However, soluble antibodies have rapid clearance and short lung retention time, limiting their effectiveness, especially for local delivery. Thus, due to the limitation represented by the emergence of multiple SARS-CoV-2 variants, it would be useful to develop other methods capable not only of capturing, but also of inactivating the virus. Among them, a successful approach might be represented by using photothermal NPs, due to the heat sensitivity of SARS-CoV-2 (Figure 15). This interesting approach finalized to capture and inactivate SARS-CoV-2 has been adopted by Cai et al. [191]. The researchers prepared a nanosystem based on neutralizing antibodies conjugated on the surface of photothermal multifunctional NPs, prepared through self-assembly of 1,2-distearoyl-sn-glycero-3-phosphoethanolamine-N [carboxy(polyethyleneglycol)-2000, NHS ester] (DSPE-PEG2000-NHS) (to form an amphiphilic polymer shell) and poly[2,6-(4,4-bis-(2-ethylhexyl)-4H-cyclopenta[2,1-b;3,4-b’]dithiophene)-alt-4,7(2,1,3-benzothiadiazole)] (PCPDTBT) (to form a semiconducting polymer core), and functionalized with an anti-SARS-CoV-2 neutralizing antibody (IgG2b), covalently attached to the NP surface by the NHS ester coupling. When irradiated by a 650 nm light-emitting diode (LED), the NPs inactivated the virus, but did not modify the capability of NPs to sequester SARS-CoV-2.

The effect of the multifunctional NPs on virus entry was demonstrated on ACE2/

HEK293T cells (HEK293T cells engineered to overexpress ACE2) infected with SARS-CoV-2 VSV (Vesicular stomatitis virus pseudotyped with the SARS-CoV-2 S protein) preincubated with NPs. Ultimately, the 650 nm LED excitation improved the inhibition efficiency of viral infection, with around three-fold enhancement in comparison with the NPs without LED irradiation. The findings were partially confirmed in vivo in K18-hACE2 transgenic mice expressing human ACE2, challenged with authentic SARS-CoV-2 and then, with the NPs administrated intranasally. Unfortunately, in these in vivo experiments photothermal treatment was not performed for technical issues.

As stated in the previous paragraph, cell-mimetic nanosystems are based on membranes of SARS-CoV-2 target cells, including macrophages. Alveolar macrophages (AM) provide the first line of defense for the host immune system against SARS-CoV-2 infection, since the virus is phagocytosed by these cells when inhaled into the alveoli, and various cytokine receptors (such as IL-6, TNF-α, or IFN-γ receptors) are expressed on their surface, facilitating their absorption. According to these concepts, multifunctional AM-like NPs with photothermal inactivation capability for COVID-19 treatment (TN@AM NPs) were developed by Li et al. [183]. PLGA cores were wrapped with AM membranes, so the resulting NPs display: (1) the viral receptor CD66a (which mediates cellular entry of the coronavirus murine hepatitis virus, MHV, and is similar to the ACE2 receptor) and (2) the cytokine receptors (CD126, which is the IL-6 receptor, and CD119 which is the IFN-γ receptor). Thus, these NPs act as a decoy, blocking coronavirus from host cell entry, and absorb various proinflammatory cytokines. Since the system was designed with the additional property to induce SARS-CoV-2 photothermal inactivation under near-infrared (NIR) laser irradiation, the NPs were doped with the NIR-absorbing organic molecule 2TPE-2NDTA [192], so that they can rapidly and efficiently convert NIR energy into the heat needed for virus photothermal disruption. Importantly, after irradiation at 808 nm for 60 min, the features of the TN@AM NPs remained unchanged. Thanks to these properties, in a surrogate mouse model of COVID-19 (using intranasal infection with the murine coronavirus MHV-A59), the intranasal treatment with TN@AM NPs coupled with NIR irradiation significantly decreased viral burden and proinflammatory cytokine levels, reducing lung damage and inflammation.

Generally, antimicrobial photodynamic therapy (aPDT) is a treatment used to inactivate or reduce the microbial population, but might be also used against viruses. aPDT is based on the employment of a non-toxic dye (photosensitizer) which, in the presence of visible light at a specific wavelength and of oxygen, produce reactive photoproducts such as ROS, damaging virus structures [193]. aPDT might be an adjuvant therapy given in addition to other drugs to treat viral infections. Moreover, it may be an effective technique for inactivation of microbes and viruses in blood or blood products [194]. Recently, Pourhajibagher et al. [195] developed PLGA NPs loaded with curcumin to be used in combination with blue laser light (450 nm) for inactivation of SARS-CoV-2 in plasma. The PLGA NPs-driven delivery allows the unfavorable physicochemical features of this polyphenol to be overcome. The effectiveness of this system plus blue laser irradiation was demonstrated on Vero cells treated with plasma containing SARS-CoV-2, while no adverse effects on plasma quality was observed. The achieved antimicrobial effects of curcumin are so potentiated by using light sources (430–450 nm wavelength range), in turn favoring the generation of ROS [196].

**Figure 16:**
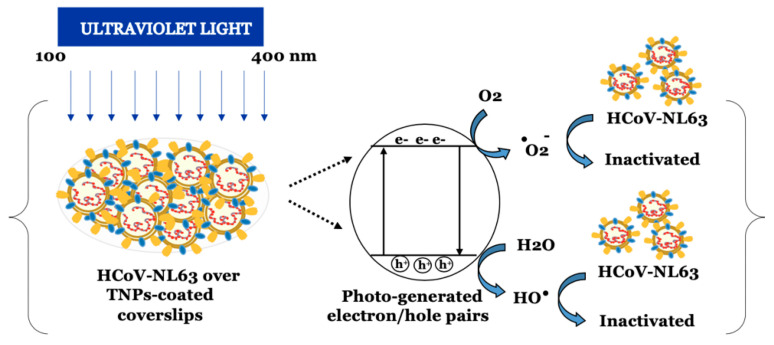
Principle of photocatalytic inactivation of the human alpha coronavirus HCoV-NL63 by UV irradiation that produces “electron-hole (e${}^{-}$-h${}^{+}$)” pairs and ROS, together with hydroxyl and superoxide radicals on the surface of TiO${}_{2}$ NPs (TNPs). Figure reprinted from Ref. [197] under the terms of the Creative Commons Attribution License.

Finally, photocatalytic properties of “self-disinfecting/cleaning” surfaces can be employed to control SARS-CoV-2 spread. Khaiboullina et al. [197] investigated the photocatalytic properties of TiO${}_{2}$ NPs as induced by the UV radiation (254 nm), towards virus deactivation (Figure 16). UV irradiation has germicidal ability and is used to reduce contamination by pathogens. Furthermore, TiO${}_{2}$ NPs are known to be effective photocatalysts with bactericidal and virucidal properties related to the production of ROS [198]. TiO${}_{2}$ NPs placed on glass coverslips plus UV-C irradiation were able to inactivate the human alpha coronavirus HCoV-NL63, which can cause acute respiratory distress symptoms. Importantly the observed virucidal effect was preserved under different humidity conditions.

### 3.6. DDSs for COVID-19 Therapy

Given the severity of the epidemic, scientists have rushed to identify antiviral strategies to combat the disease. There are several strategies for discovery of anti-COVID-19 drugs for coronaviruses, including empirical testing of known antiviral drugs, repurposing of old drugs used for non-antiviral properties, large-scale phenotypic screening of compound libraries, and computational studies. A fundamental issue in these studies is represented by the development of opportune delivery systems, which are able to ameliorate and optimize drug pharmacokinetic and pharmacodynamic properties, and, in particular, obtain drug therapeutic concentrations in the target organ(s) using non-invasive administration routes and minimizing the risk of adverse reactions.

Nanocellulose has emerged as a new class of biomaterials with promising potential application in the filtration of viruses. Nanocellulose is uniform in diameter and has excellent nanofibrillar morphology [199]. Nanocelluloses are also useful ingredients of DDSs due to their biocompatibility and biodegradability features, and they were previously investigated as carriers for various types of antivirals [200]. Recently, NPs obtained via the dispersion of crystalline nanocellulose (CNC) in curcumin/polyvinyl alcohol (PVA) aqueous medium (CNC/PVA/curcumin NPs) have been used against SARS-CoV-2 [201]. The synthesized nanosystem is interesting for the unique physico-chemical properties of the sulphuric acid hydrolyzed nanocellulose and the well-known antiviral properties of curcumin. The sulfuric acid hydrolyzed nanocelluloses show a negative surface charge, so they can be well dispersed in aqueous solutions. Importantly, the sulphuric acid hydrolyzed CNC surfaces, which have negatively charged groups, are responsible for electrostatic interaction with the positively charged SARS-CoV-2 spike proteins. The binding of CNC with SARS-CoV-2 spike can prevent the interaction of the virus with host ACE2 protein and its cell penetration. Since the system was designed for inhalation therapy, drug release was measured in an acidic medium at a pH similar to that of mucosal airway [202]. This is an additional advantage, since curcumin is stable in an acidic medium but unstable in neutral or alkaline environments.

The infectivity of coronaviruses is also related to the lipid composition of host cell membranes, which are rich in cholesterol and sphingolipids. The latter are involved in coronavirus cell penetration; in particular, the ACE2 receptors are concentrated at the level of lipid rafts of the host cell membrane, playing a key role in SARS-CoV-2 infection [203]. Recently, Paolacci et al. [204] explored the potential anti-COVID-19 effect of α-cyclodextrin through an in silico study, considering its interaction with Spike and ACE2 proteins in combination with hydroxytyrosol. The starting point of this study is that α-cyclodextrins are natural cyclic oligosaccharides and excellent lipid exchangers, able to scavenge phospholipids from the plasma membrane and so change membrane lipid composition. Furthermore, α-cyclodextrins can interfere with the endocytosis pathway through a mechanism related to the phosphatidyl-inositol system [205].

A small natural polyphenol which has antiviral activity is hydroxytyrosol [103], which can be captured in the hydrophobic cavity of α-cyclodextrins [206]. The study evidenced that α-cyclodextrin interacts with Spike and ACE2 proteins; moreover, hydroxytyrosol interacts with Spike and ACE2 proteins, even if α-cyclodextrin results a better inhibitor than hydroxytyrol against ACE2 protein. Finally, the complex α–cyclodextrin–hydroxytyrosol is able to interact with both Spike and ACE2 proteins, as its binding energy with ACE2 is very similar to that of α-cyclodextrin. Lipid–polymer hybrid systems are considered promising carriers of some drugs, such as the baricitinib (BTB), whose bioavailability is very low due to high solubility and poor permeability. BTB is a drug generally used for rheumatoid arthritis therapy, but now approved for COVID-19 treatment. To overcome its pharmacokinetic limitations, Anwer et al. [207] proposed the synthesis of BTB-loaded hybrid NPs based on lipids (stearin) and polymers (PLGA) using the single-step nanoprecipitation method. These nano-hybrid systems have shown improved BTB bioavailability in comparison to the normal suspension of pure BTB after oral administration in rats; thus, these systems are potentially interesting for a sustained release profile of BTB.

A lipid polymer hybrid (LPH) nanoformulation was proposed by Khater et al. [208] to successfully deliver the selective serotonin reuptake inhibitor (SSRI) fluoxetine hydrochloride (FH). SSRIs are antidepressant drugs which could be repurposed against COVID-19 [209,210]. Through in silico studies, Khater et al. showed that FH can effectively bind with SARS-CoV-2 main protease via hydrogen bond formation. So, to improve its efficacy, FH was loaded in LPH NPs based on PLGA, lecithin soybean, and tween 80. The FH loaded within LPH NPs improved cellular internalization in human lung fibroblast (CCD-19Lu) cells. As is well known, the control of the polymeric matrix morphology loaded with metal NPs is fundamental to improve the drug loading and release. A new nanocomposite based on the spinel ferrite ZnFe${}_{2}$O${}_{4}$ and halloysite was developed by Jermy et al. [211] to be used as pulmonary nanocarrier. Halloysite (Al${}_{2}$Si${}_{2}$O${}_{5}$(OH)${}_{4}$·2H${}_{2}$O) is a natural mineral clay similar to kaolin [212] and zinc ferrite; it has several biomedical applications, including drug delivery [213]. The nanocomposite was functionalized with dexamethasone (DEX, used in the therapy of patients with COVID-19) and coated with PEG using a lyophilization technique. The particle size of the nanocomposite (in the range 50–70 nm) and the PEG coating facilitate the penetration of the system in tracheal mucus. The penetration of SARS-CoV-2 into the cell is a pH dependent process, since the interaction of the virus with ACE2 receptor occurs at pH 5.5 [214]. Interestingly, the release of DEX from ZnFe${}_{2}$O${}_{4}$/Hal/DEX/PEG was also found to depend on the pH conditions, since the carrier is sensitive to pH 5.6; this property is related to the PEG addition to the composite material. These findings led us to hypothesize a successful employment of this system for pulmonary drug delivery in lung diseases and in particular, as it is a pH-sensitive system, in COVID-19.

As reported in the previous section, there is a large amount of interest in the possible development of Zn-based drugs for therapeutic and prophylactic treatment of COVID-19 [215]. There is still not enough evidence to consider Zn administration or supplementation really promising against COVID-19 infection, especially in Zn-deficient patients [114,216]. However, Zn could be employed to improve the performance of other antiviral agents. Ghareeb et al. [217] combined ZnO NPs with berberin (BER), an isoquinoline alkaloid with antimicrobial activity and antiviral properties [218], by absorption of cationic BER molecules on the surface of negatively charged ZnO NPs. *In silico* analyses demonstrated that the ZnO BER complex binds to PLpro (through O with O-atom in Asp77 and Glu78), to spike protein (through its O atom with O-atom of Ile666 and Pro862) and to the spike protein RBD (through hydrophobic interactions). These results, confirmed through biochemical assay, indicate that ZnO BER complex acts as anti-COVID-19 by inhibiting the virus’ entry through interactions with the ACE2 enzyme on the cell surface and blocking the spike protein interaction and PLpro activity. Similarly, ZnO NPs functionalized with triptycene (a rigid and highly symmetric organic molecule widely used for the synthesis of DDSs) and impregnated with the ellagic acid (a polyphenol endowed with antiviral activity) showed direct inactivation, via a virucidal mechanism, of the human coronavirus 229E, belonging to the same family as SARS-CoV-2 and causing respiratory disorders [219].

For instance, the capability of azithromycin (AZT), a second-generation macrolide with broad spectrum antibacterial activity, to induce a reduction of ACE2 expression in vitro human airway cell is increased by the addition of ZnSO${}_{4}$ [220]. Additionally, the use of computer simulation showed that AZT-Zn${}^{2+}$ complexes can effectively prevent the duplication and assembly of SARS-CoV-2, so that the combined use of AZT with Zn salt might have both prophylactic and therapeutic applications in COVID-19.

Remdesivir is approved for treatment of COVID-19. Unfortunately, the drug must be administered intravenously and has several unfavorable issues, including hydrophobicity, rapid hydrolysis to a hydrophilic metabolite, and instability in aqueous solution, which limit its efficacious employment. A theoretical study (based on combining molecular docking with dissipative particle dynamics simulations) was carried out by Wu et al. [221] to design PLGA NPs loaded with remdesivir and grafted with the ACE inhibitor (ACEI) lisinopril; the synergistic effect of lisinopril and remdesivir (due to the action on two main targets) may be fundamental. Specifically, remdesivir is loaded in the hydrophobic PLGA core and lisinopril in the thin hydrophilic shell. For this purpose, the nanosystems proposed by Wu et al. might be useful for efficient co-delivery of hydrophilic and hydrophobic agents in COVID-19 treatment. For the same purpose, Vartak et al. [222] formulated stable aerosolized nanoliposomes made of cholesterol and 1,2-distearoyl-sn-glycero-3-phosphoethanolamine-N-[amino(polyethylene glycol)-2000] (DSPE-PEG2000), together with ( 1,2-dipalmitoyl-sn-glycero-3-phosphocholine (DPPC) and/or 1,2-dioleoyl-sn-glycero-3-phosphocholine (DOPC). These systems allow the preparation of liquid liposomal formulations that can be used effectively through nebulization, allowing efficient and more comfortable drug delivery of the loaded remdesivir.

A particularly innovative approach to treat COVID-19 is the siRNA-NP therapy proposed by Idris et al. [223]. Small interfering RNAs (siRNAs) are short double-stranded RNA molecules that induce gene silencing at the transcriptional or post-transcriptional level. In detail, Idris et al. developed several siRNAs targeting highly conserved virus regions (particularly the RdRp), and two of these siRNA (siHel2 and siUC7) exhibited effective inhibition of the virus (>90%). These siRNA were encapsulated into stealth LNPs based on DOTAP (1,2-dioleoyl-3-trimethylammonium-propane) and MC3 (DLin-MC3-DMA;6Z,9Z,28Z,31Z)-heptatriaconta-6,9,28,31-tetraen-19-yl-4-(dimethylamino) butanoate), which have shown enhanced efficiency in targeting the lung [224]. The ability of the DOTAP/MC3 LNP-siRNA to deliver functionally repressive siRNA was demonstrated through intravenous injection in K18-hACE2 mice intranasally infected with SARS-CoV-2. COVID-19 infection can cause multiple side effects that contribute to the onset of acute kidney injury (AKI). Kidney and glomerular cells contain ACE2 receptors and are susceptible to SARS-CoV-2 virus entry and infection. Surnar et al. [225] developed orally administrable, dual-targeted NPs to deliver the antiviral drug IVM, and the antioxidant supplement coenzyme Q10 (CoQ10), with the aim to treat SARS-CoV-2 infection and AKI related to COVID-19. These polymeric NPs are based on PLGA-b-poly(ethylene glycol)-maleimide (PLGA-b-PEG2000-Mal) conjugated with Fc (fragment crystallizable) and GLU (a glutamate–urea-based ligand). Sulfhydryl immobilization was used to conjugate Fc fragments onto the NPs. The Fc fragment targets the neonatal Fc receptor (FcRn, which mediates immunoglobulin transport across the epithelium in a pH-driven way) for transport of NPs to the bloodstream through the gut endothelial barrier [226]. GLU targets the prostate-specific membrane antigen (PSMA) expressed on kidney proximal tubules, especially following kidney diseases and damage [227]. The efficacy of the system in crossing multiple barriers and counteracting viral infection was tested in an in vitro barrier model with Caco-2 cells on the apical side and HEK293T cells on the basolateral side; the human colorectal adenocarcinoma Caco-2 cells (expressing the FcRn receptor needed for the transcytosis of the Fc-conjugated NPs) mimic the gut epithelial barrier, while the human embryonic kidney HEK293T cells (expressing higher PSMA levels on their surface) mimic the kidney. Finally, the good drug biodistribution following oral administration was verified in BALB/c mice. The evidence of Surnar et al. shows that this dual-targeted system developed to treat COVID-19 is a useful proof-of-concept nanoplatform for future treatments of renal complications related to the viral infection.

## 4. Computer-Based Investigations for Discovery of Plant-Derived Drugs as Potential Anti-SARS-CoV-2 Agents: Focus on Terpenes and Terpenoids

To choose the molecules likely to be promising candidates for anti-COVID-19 treatment, fundamental help is given via the employment of high-performance-computing-based tools. These techniques are indispensable for medicine screening to accelerate the development of specific drugs against COVID-19, and might take significant advantage of the knowledge of the structure of the possible targets (in our case, at the level of the host cell), to which the potential drug can competitively bind. In particular, molecular docking calculations are based on receptor active site regions to search for whether ligands interact with the target structure and the optimal binding mode between them; MD simulation is a method that simulates experimental conditions and can display the microscopic evolution of the system at an atomic level [26,228].

As stated in the previous paragraphs, blocking the penetration of the virus into host cells therefore continues to represent a fundamental strategy on which research efforts into prevention of COVID-19 infection are focused. It is also evident that each therapeutic treatment targeting the viral proteins might be less efficient at hampering the worldwide spread of the different SARS-CoV-2 variants responsible for more infectivity and mortality in comparison with the original Wuhan strain. This evidences the importance of developing new approaches to block the entry of all SARS-CoV-2 variants into the host cells, targeting cell proteins which may strongly contribute to virus cell penetration. The ACE2 receptor protein is not the only gateway present on the cell membrane to allow the entry of this virus into cells; but other cell surface proteins capable of playing this role have also been identified. Among these proteins is the GRP78 [229], a heat shock protein located in the lumen of the endoplasmic reticulum (ER). There is widespread evidence that this protein can favor the entry of various viruses into the cell. Many research studies on anti-SARS-CoV-2 agents are based on the idea that ACE2 is the primary receptor of SARS-CoV-2 and GRP78 is a secondary receptor used by the virus, especially when it is overexpressed [230,231]. GRP78 is the main chaperone protein of the unfolded protein response.

In fact, when this protein is overexpressed, it can escape the KDEL motif receptors responsible for its retention in the ER, due to saturation or downregulation of the receptors, and translocates to the cell membrane [232,233] acting as a cell surface receptor susceptible to virus recognition via its substrate binding domain (SBD). Thus, it is potentially useful for the entry of the virus into the cells. In particular, ER stress conditions, inducible by hypoxic or glucose deprivation conditions, cause overexpression of GRP78 genes and increased production of GRP78 [61]. Furthermore, GRP78 is expressed in adipocytes and is a potential target for a therapeutic approach to obesity [234,235], a condition recognized as a risk factor for severe COVID-19 outcomes [236,237,238].

Over the last two years, one of the most investigated classes of natural compounds as potential anti-COVID-19 agents has been terpenes and terpenoids. Widely found in nature, these compounds constitute one of the most important classes of natural or secondary metabolites, with over 50,000 compounds isolated from plants so far. They derive from mevalonic acid and consist of five carbon isoprene units assembled with each other. While terpenes are simple hydrocarbons, terpenoids—divided into hemiterpenoids, monoterpenoids, homoterpenoids, sesquiterpenoids, diterpenoids, sesterpenoids, triterpenoids, tetraterpenoids, and polyterpenoids, depending on the number of carbon atoms—are modified terpenes containing different functional groups and oxidized methyl [239]. Terpenoids play important roles in the interaction between plants and environment and insects, acting as chemoattractants or chemorepellents and participating in plant defense systems (some are used as pesticides) [240].

Many terpenoids exhibit various pharmacological properties, including anti- inflammatory, antitumoral, analgesic, antibacterial, antifungal, and antimalarial properties [241,242,243]. Moreover, various studies have also reported the antiviral potential of some of these compounds. Since the COVID-19 pandemic outbreak, numerous in silico investigations have been conducted to spot terpenoids against several steps of the SARS-CoV-2 life cycle; this review takes into consideration only the studies proposing an efficient interaction, as shown by computer-based techniques, between terpenoids and the target proteins ACE2 (on the host cell) and Spike (on the virus), the main players in the virus entry process. In addition, since the importance of other host proteins for virus cell penetration, such as GRP78, has recently been clearly underlined, but few data have been reported in the literature regarding the possible interaction between terpenoids and this protein, here, we are reporting preliminary in silico investigations on the herein reviewed terpenes/terpenoids to identify possible inhibitors of the spike/GRP78 interaction.

### 4.1. Terpenes and Terpenoids as Antivirals

Terpenes were shown to be active against herpes simplex virus-1 (HSV1), hepatitis C virus (HCV), and human immunodeficiency virus (HIV). Antiviral activity against HSV1 has been reported for various essential oils extracted from South American plants containing monoterpenes, such as carvone, β-pinene, camphor, and carveol [244], and from Lebanese plants containing α- and β-pinene, and 1,8-cineole [245]. Anti-HSV-1 activity, with a reduction in viral load, has also been reported for monoterpenes such as α-pinene and α-terpineol from essential oils extracts isolated from eucalyptus, tea tree, and thyme. In particular, the authors reported the higher antiviral efficacy of the mixture of monoterpenes versus single monoterpenes due to its lower toxicity against host cells [246]. Anti-HSV-1 activity has also been reported for the monoterpenoids isoborneol [247], terpinene, α-terpineol, and thymol [246], and for the triterpenes morinic acid and betulonic acid from *Rhus javanica* [248], whereas Brezani et al. [249] reported the antiherpetic activity of tereticornate A from *Eucalyptus globulus*, with activity even higher than acyclovir against HSV-1 replication. Inhibition of viral entry of HCV has been reported for saikoponins from the roots of *Bupleurum kaoi* [250], and for the monoterpene loliolide from *Phyllanthus urinaria* [251]. Anti-HCV activity has been also reported for artemisinin and artesunate, which are mainly known for their antimalaria activity [252]. HIV activity has been reported for various terpenoids, such as glycyrrhizin [253], the pentacyclic triterpenoids betulinic acid from *Syzigium claviforum*, which inhibits fusion, reverse transcriptase, and assembly of the virus [254,255], and oleanolic acid and its derivatives, capable of inhibiting HIV-1 protease activity [256].

Since 2003, following the SARS emergency, numerous phytocompounds, including many terpenes and terpenoids, have been screened for their antiviral activity against coronaviruses. Wen et al. [257] evaluated the activity of 221 phytocompounds against SARS-CoV using a cell-based assay measuring SARS-CoV-induced cytopathogenic effect on Vero E6 cells. Among these phytocompounds, they identified ten diterpenoids (abietane type and labdane type), two sesquiterpenoids, and two lupane-type triterpenoids as potent inhibitors with good cytopathogenic effects. Moreover, savinin and betulinic acid possess inhibitory effects on SARS-CoV 3CLpro activity with a competitive inhibition mode of action. Park et al. [258] identified diterpenoids tanshinones from *Salvia miltiorrhiza* as non-competitive specific inhibitors of the SARS-CoV 3CLpro and PLpro, with cryptotanshinone, tanshinone IIA, and dihydrotanshinone I showing the most potent inhibitory activity, very likely related to the presence of the dimethyl tetrahydronaphthalen structure. Chang et al. [259] extracted 22 triterpenoids from *Euphorbia neriifolia* L. leaves and tested their activity against human coronavirus (HCoV) strain 229E. One of these triterpenoids, 3β-friedelanol, possesses potent anti-HCoV activity, suggesting that the friedelane skeleton could represent a potential scaffold for developing new anti-HCoV drugs. Ryu et al. [260] studied the 3CLpro inhibitory activity of the four quinone-methide triterpenes celastrol, pristimerin, tingenone, and iguesterin, isolated from *Tripterygium regelii*. These compounds showed potent inhibitory activity, related to the presence of a quinone-methide moiety in A ring and a more hydrophobic E-ring.

Antiviral activity of glycyrrhizin against SARS-CoV has also been reported. Cinatl et al. [261], in fact, reported the antiviral activity of glycyrrhizin in Vero cells infected with two clinical isolates of coronavirus from patients with SARS-CoV. Glycyrrhizin inhibited SARS-CoV adsorption, penetration, and replication by inducing nitrous oxide synthase activity, and so increasing intracellular levels of nitric oxide. The activity of glycyrrhizic acid against SARS-CoV-2 has also been recently reported [262,263]. Yu et al. used a NanoBit assay, and reported that glycyrrhizic acid disrupts SARS-CoV-2 S RBD/ACE2 interaction at low concentrations (IC${}_{50}$ = 22 μM) [262], whereas Li et al., using an S protein-pseudotyped lentivirus and through surface plasmon resonance, showed that it interferes with S protein binding to host cells [263].

### 4.2. In Silico Studies on Terpenoids Interaction with ACE2 and SARS-CoV-2 Spike Protein

The interactions between terpenoids and SARS-CoV-2 Spike protein and ACE2 have been investigated by several authors in in silico studies. Ahmad et al. [264] performed an in silico screening that started with a ligand-based similarity search that filtered 40 FDA-approved compounds in DrugBank as analogs of glycyrrhizin. Glycyrrhizin has been extensively reported for its anti-SARS-CoV-2 potential [261,265,266,267,268] and has been used as a positive control in the simulations described by the authors. The 40 retrieved compounds were then docked against ACE2, by means of a consensus approach that used two different docking software, i.e., AutoDock [269] and DockThor [270]. The three most interesting compounds from the docking analyses, (deslanoside, digitoxin, and digoxin; see supplementary information) were further investigated in silico. MD simulations, performed using AMBER18 [271], confirmed the stability of the deslanoside/ACE2, digitoxin/ACE2, and digoxin/ACE2 complexes. Estimation of the binding free energies was carried on by MMGBSA and MMPBSA, which gave similar energy evaluations and highlighted the strong binding affinity of the simulated ligands toward ACE2. The interaction of digitoxin with ACE2, as well as with S protein, was also confirmed through an in silico study by Kalhor et al. [272].

Emon et al. [273] performed virtual screening of the bioactive constituents from tea, prickly chaff, catechu, lemon, black pepper, and synthetic compounds using Autodock Vina. Among these constituents, three terpenes, i.e., limonene, sabinene, and pinene, were docked against ACE2 (PDB IDs: 6VW1, 1R42). The docking score of the three compounds ranged from −4.5 to −4.8 kcal/mol and, compared to the other bioactive compounds analyzed in the same work, it was not good enough to consider them as potential ACE2/SARS-CoV-2 S inhibitors. Gyebi et al. [274] screened 106 bioactive terpenoids from African medicinal plants through molecular docking analysis against hACE2 and SARS-CoV-2 S protein using AutoDock Vina. The results revealed two pentacyclic terpenoids (24-methylene cycloartenol and isoiguesterin) interacting with the hACE2 binding hotspots for the SARS-CoV-2 S protein, while 3-benzoylhosloppone and cucurbitacin B interacted with the RBD and S2 subunit of SARS-CoV-2 spike protein, respectively. The best docked solutions were further studied by MD simulations, using NAMD, that confirmed the stability of the complexes, and MM-GBSA binding free energy decomposition calculations that corroborated the docking interactions [275].

Muhseen et al. screened a dataset of 1000 plant bioactive terpene compounds with reported therapeutic potential retrieved from the medicinal plant drug databases NPACT [276] and MPD3 [277], through rigid and flexible docking simulations using Autodock Vina against the SARS-CoV-2 S protein [278]. Out of 1000 terpenes, 3-β-O-[α-L-rhamno pyranosyl-(1->2)-α-L-arabinopyranosyl]olean-12-ene-28-O-[α-L-rhamnopyranosyl-(1-> 4)-β-D-glucopyranosyl- (1-> 6)- β-D-glucopyranosyl] ester (NPACT01552), 3-O-[β-D-gluco pyranosyl (1->3)-β-D-galactopyranosyl(1->2)]-β-D-glucuronopyranosyl-oleanolic acid-28-O-β-D-glucopyranoside (NPACT01557), and glycyrrhizin (NPACT00631) showed the most favorable binding energies (−11 kcal/mol, −10.3 kcal/mol and −9.5 kcal/mol, respectively) when interacting with the RBD. Submitted to GROMACS [279] MD simulations, these three terpenes showed stable bound conformations and free energy MMPBSA calculations, and confirmed the favorable interactions predicted by the docking simulations. Through energy decomposition analyses, the authors identified L455, F456, A475, F486, and Y489 as ligand/target interaction hotspots.

Autodock [269] was used by our research group [228] for in silico screening of a series of terpenoids, such as triterpenoids and limonoids, against the SARS-CoV-2 RBD. These compounds were selected for their easy availability from different natural sources of the Mediterranean area, as well as from very common agro-industrial wastes. None of the simulated compounds appeared to be effective in binding to the S protein RBD, since, despite the reported good Autodock-predicted binding energy (glycyrrhizin −9.25 kcal/mol and masticadienoic acid −8.42 kcal/mol), they lost contact with the amino acid residues during 120ns-long Desmond [280] MD simulations. Aminu et al. [281] screened twelve terpenes against SARS-CoV-2 S protein using the Vina software. Among the docked compounds, Rediocide A and phytolaccoside B showed the highest binding affinities toward the protein, i.e., −8.0 kcal/mol and −7.1 kcal/mol, respectively.

Phytochemicals from essential oils, extracted from plants belonging to families such as *Lamiaceae, Lauraceae, Myrtaceae, Apiaceae, Geraniaceae*, and *Fabaceae*, were screened in silico against SARS-CoV-2 S protein by Kulkarni et al. [282], who even tried to correlate biological activities with density-functional-theory-based molecular descriptors. In this study, docking was performed using Autodock vina in PyRx virtual screening open source software. The docking score of the screened terpenes and terpenoids ranged from −4.8 to −5.4 (kcal/mol), and carvacrol, geraniol, and L-4-terpineol displayed better predicted binding affinity compared to the other ligands. Yepes-Pérez et al. [283] evaluated the potential antiviral properties of the components of the medicinal herb *Uncaria tomentosa* (cat’s claw) focusing on the binding interface of the SARS-CoV-2 S-RBD and ACE2 (PDB ID: 6M17 [284]) and the viral S protein (PDB ID: 6VYB [285]) using AutoDock Vina. Among the bioactive constituents, polyhydroxylated triterpene uncaric acid emerged for its predicted binding affinity (−7.0 kcal/mol) for the RBD/ACE2 interface, as well as for its interaction stability, which was challenged by MD simulations. Liquorice (*Glycyrrhiza* species) has been recommended by traditional Chinese medicine in the treatment of infections caused by SARS-CoV-2, thus making its components widely investigated for this therapeutic indication. Sinha et al. [286] performed a molecular docking simulation study of *Glycyrrhiza glabra* bio-constituents against SARS-CoV-2 S protein (PDB ID: 6VSB) using Autodock Vina [287]. Glycyrrhizic acid exhibited the best docking results among all the ligands, with predicted binding energy of −9.2 kcal/mol. The stability and the favorable binding free energy of the spike-bound glycyrrhizic acid was supported by all-atoms MD simulations and MMPBSA calculations.

Similarly, Yi et al. [288] screened 125 compounds from the Chinese herbal medicine licorice using Autock Vina in a two-step workflow. The authors report that among the investigated terpenoids, glycyrrhetinic acid, 3-O-β-D-glucuronosyl-glycyrrhetinic acid, and licorice-saponin A3 showed remarkably higher affinities compared to glycyrrhizic acid, which had been reported as a SARS-CoV-2 inhibitor [262]. The inhibition of the spike/ACE2 binding was confirmed by ELISA: at 10 mM, glycyrrhetinic acid, 3-O-β-D-glucuronosyl-glycyrrhetinic acid, and licorice-saponin A3 exhibited inhibition rates of 51.9%, 50.2% and 45.1%, with a IC${}_{50}$ values of 10.9, 14.1 and 8.3 mM, respectively. The dose-dependent activities of glycyrrhetinic acid and licorice-saponin A3 were confirmed in two cellular models (Vero E6 and Caco-2 cells) of SARS-CoV-2 infection. Through SPR and ELISA experiments on mutant proteins, the authors proved that the S protein residue Y453 is fundamental for the binding of both glycyrrhetinic acid and licorice-saponin A3.

### 4.3. In Silico Studies on Terpenoids Interaction with GRP78 Protein

As stated before, GRP78 was also identified as a receptor for SARS-CoV-2 S protein that enables entry into cells by improving virus attachment. Regarding that, Quimque et al. [289] performed the first molecular docking experiment that targeted the binding domain of the S protein to GRP78, which is located in the Region IV (C480–C488) of the S protein. Several antiviral secondary metabolites from fungi (97 compounds) were screened, using the UCSF Chimera platform, and the terpenoid 11a-dehydroxyisoterreulactone A exhibited high binding affinity (−10.5 kcal/mol) on the S binding domain with GRP78. The stability of the ligand–protein complex was supported by MD simulations. Ibrahim et al. [60] investigated GRP78 as receptor for SARS-CoV-2 S-glycoprotein using molecular modeling studies. The authors performed combined molecular modeling and structural bioinformatics studies to predict the COVID-19 S binding site to the cell-surface receptor (GRP78). The docking revealed that the binding might be more favorable between regions III (C391–C525) and IV (C480–C488) of the S protein model and GRP78, but Region IV, composed of nine key residues (ILE426, THR428, VAL429, VAL432, THR434, PHE451, SER452, VAL457, and ILE459), is the main driving force behind GRP78 binding. Ghasemitarei et al. [290] applied MD simulations to study the effect of oxidation of the highly reactive cysteine (CYS) amino acids of RBD on its binding to ACE2 and GRP78, and their results were in agreement with the previously cited work. Similarly, Allam et al. reported molecular docking studies of potential inhibitors comprising small molecules and peptides capable of blocking the recognition of the cellular GRP78 receptor by the viral S protein [291]. Docking studies were performed on two binding domains in GRP78, the nucleotide-binding domain (NBD) that hosts Adenosine triphosphate (ATP) and the SBD characterized by the residues identified in previous works [60]. Inhibition of the ATP binding site disrupts the functional cycle of the protein by changing the protein conformation, which can lead to inhibition of viral entry. So, due to the paucity available in the literature regarding this innovative topic, we performed in silico investigation of the above reviewed 24 compounds as potential inhibitors of GRP78/spike interaction using the Autodock software on both NBD and SBD (see Materials and Methods in the supporting information). Molecular docking simulations were performed on the structure of GRP78 (PDB ID:5E84 [292,293,294]), centering the two docking grids on the experimental bound conformation of ATP for the NBD and on residues Ile426, Thr428, Val429, Val432, Thr434, Phe451, Ser452, Val457, and Ile459 for the SBD, according to the study by Ibrahim et al. [60].

**Figure 17:**
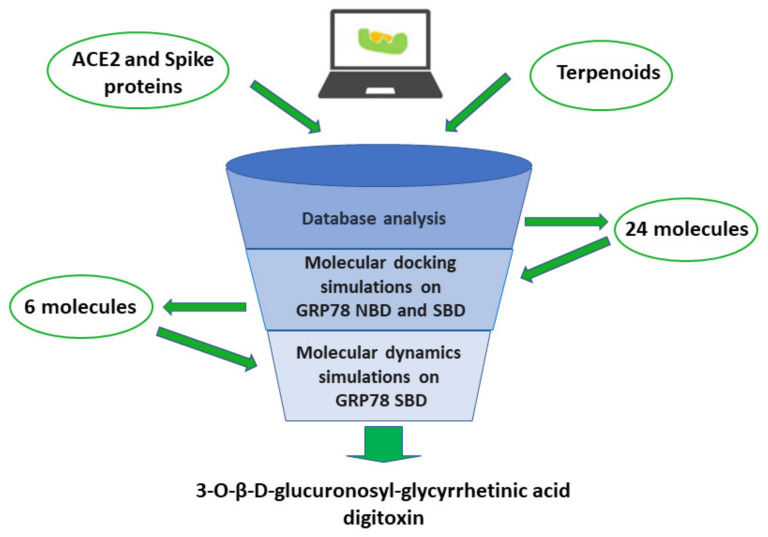
Starting with the examination of scientific literature, molecular docking and molecular dynamics simulations have allowed the identification of two terpenoids (digitoxin and 3-O-β-D-glucuronosyl-glycyrrhetinic acid) as potential ligands of GRP78 SBD.

Docking simulations at the NBD did not result in any favorable predicted binding affinity, ranging between −5.1 and 1.3 kcal/mol. Based on these results, no further investigation was carried out on NBD. In opposition, encouraging results were obtained from docking on GRP78 SBD (Table S1). The six best ranked compounds, whose docking scores ranged between −7.15 and −6.24 kcal/mol, were digitoxin, masticadienoic acid, 3-benzoylhosloppone, 3-O-β-D-glucuronosyl-glycyrrhetinic acid, isoiguesterin, and glycyrrhizin. These latter compounds were subjected to MD simulations (see materials and methods in supporting information) in order to evaluate the stability of the predicted GRP78/ligand complexes and the amino acid residues involved in protein/ligand interactions (Figure 17).

We used the TIP3P water model [295] in a periodic boundary conditions orthorhombic box. Systems were neutralized by Na${}^{+}$ and Cl${}^{-}$ ions, which were added until a concentration of 0.15 M was reached. The default six-stages relaxation protocol distributed with Desmond [296] was run prior to the production stage. MD simulations analyses plots of the six best ranked compounds are reported in the supporting information. Briefly, the 3-benzoyl-hosloppone/GRP78 complex showed high values of ligand RMSD (root mean square deviations) and RMSF (root mean square fluctuations) during the 48 ns long MD simulation, resulting in the loss of contact with amino acid residues of the protein (Figure S2). Masticadienoic acid, despite showing slightly more stable RMSD values in the first 40 ns of MD simulation, did not show stable protein/ligand contacts in agreement with the high ligand RMSF (Figure S3). Similar results were found through the MD simulation of isoiguesterin (Figure S4). More interesting results were instead obtained for digitoxin, which showed stable RMSD values during the 48 ns of MD simulation and stable interactions with GRP78, in particular with ILE450, SER452, and THR458 (Figure S5). So, our findings evidence that digitoxin could inhibit SARS-CoV-2 cell penetration acting on several targets, the host proteins GRP78 and ACE2 as well as the viral S protein [270,272]. In agreement with this hypothesis, digitoxin, as well as other cardiac glycosides, has been reported to possess antiviral properties against some RNA viruses and DNA viruses [297,298]. Recently, Caohuy et al. [299] reported that digitoxin can potently inhibit ACE2 binding to SARS-CoV-2 S (which was also found when mutant spike variants were tested) and block virus penetration and infectivity in human lung cells. In addition, digitoxin can inhibit the TNFα-dependent proinflammatory response to influenza virus infection [47], and has also been reported to be a potent and efficacious suppressor of TNFα-dependent proinflammatory cytokine expression in cystic fibrosis patients [300], and this antinflammatory activity may be significantly useful in COVID-19 patients. Unfortunately, cardiac glycosides used to treat patients with heart failure have a narrow therapeutic index, and this safety aspect needs to be considered when proposing digitoxin as a potential anti-COVID-19 agent. However no toxicity or adverse events have been reported when digitoxin was administered to patients with cystic fibrosis [300]. Furthermore, the digitoxin scaffold might be further investigated as a GRP78 inhibitor.

MD simulations of the glycyrrhizin/GRP78 complex showed pretty unstable ligand RMSD values and, looking at the recorded ligand RMSF values, much of the instability is probably due to the high fluctuation of the disaccharide portion (Figure S6); in fact, the removal of one of the two sugars, to provide the 3-O-β-D-glucuronosyl-glycyrrhetinic acid, stabilizes the RMSD and RMSF values, (Figure S7) and facilitates the formation of stable and favorable protein/ligand contact with residues ILE450, SER452, and THR458. Interestingly, the 3-O-β-D-glucuronosyl-glycyrrhetinic acid, as previously reported [288] in the ”blocking” assay, showed remarkable antiviral activity, with EC50 values of 5.37 M. On the basis of our findings and of the data reported in literature (Yu et al. [262] and Yi et al. [288]), it is evident that this molecule should be further investigated via biological screening and biophysical experiments to confirm its possible “dual” target molecular mechanism (Spike/GRP78 binding) in the inhibition of SARS-CoV-2 cell penetration.

## 5. Conclusions and Future Perspectives

In April 2022, the members of the World Health Organization (WHO) Emergency Committee for COVID-19 meeting in Geneva unanimously agreed that COVID-19 is still a “PHEC” (Public Health Emergency of International Concern), as the pandemic constitutes an extraordinary event that continues to negatively affect the health of populations all over the world. As pointed out by WHO, SARS-CoV-2 remains a new respiratory pathogen with unpredictable viral evolution, aggravated by widespread circulation, intense transmission in humans, and appearance of multiple SARS-CoV-2 VOC (characterized by increased transmissibility and/or virulence, or reduced effectiveness of countermeasures). Total cases of contagion since the start of the pandemic have exceeded 500 million worldwide, with over 6 million deaths [1].

Although the scientific community has been caught unprepared by the health emergency due to the SARS-CoV-2 pandemic, in the face of this new virus and an unknown and exceptionally serious disease, its response has been broader than ever, in terms of rapid sharing of discoveries as well as organizational initiatives and proposals.

Our review aimed to describe the contribution that nanoscale technologies can make in the fight against COVID-19 throughout various fields: on the one hand, the design of innovative materials useful as viral spread control measures and the discovery of new drugs capable of specifically targeting the virus or host cell, and on the other hand, the use of computer-based technologies (such as molecular docking and dynamics analysis) which allow results to be obtained, and therefore provide answers in a very short time. This a fundamental issue in scenarios that have proved to be unpredictably changeable, such as that of COVID-19. Unlike other papers in the literature, we have taken into consideration only research that, in various aspects, concern the specific activity towards SARS-CoV-2, and not generic and/or non-specific activity against respiratory viruses or other coronaviruses. This reflects the large amount of results that the scientific community has produced in the last two years, interpreting the need to have specific weapons to face a pandemic of extraordinary duration, diffusion, and gravitas.

Among those examined in this review, nanosystems appear particularly interesting for capturing and inactivating the virus, even through the simultaneous use of photothermal and photocatalytic treatments. Some nanomaterials have been developed for use via inhalation/nebulization; this is an undoubted advantage, as it is a non-invasive drug delivery method providing drug therapeutic concentrations specifically at the level of the main route of virus penetration and the disease target organ, with a decreased risk of systemic effects. On the other hand, the development of nanodecoys/nanosponges/nanocatchers, containing the host cell proteins involved in the infection, is certainly an innovative method that could allow us to overcome the problem of the numerous emerging viral variants, but also counteract the symptoms characterizing the infection (e.g., overinflammation). Furthermore, the molecules capable of targeting GRP78, identified by computer-based methodologies, not only represent new therapeutic candidates capable of inhibiting the cellular penetration of the virus through the ACE2 pathway, but can also provide an answer to the therapeutic need of a subgroup of patients, i.e., obese or overweight subjects, since this protein is also expressed in adipocytes and appears to be overexpressed in obese patients [235]. In addition, as evident from the studies we have reported, in silico investigations also represent a solid starting point for the development of various types of anti-COVID-19 nanomaterials.

It is obvious that the results obtained so far by the scientific community in relation to COVID-19, and the employed research strategies and technologies, could be extrapolated and taken into consideration for other new emerging viruses. However, at this stage of the research, the translation of the findings from the laboratory to industrial and clinical fields appears to be very problematic and still far from a possible implementation. The industrial methodologies employed to realize these materials are extremely complex and sophisticated, highlighting the presence of several important obstacles in terms of reproducibility, scale-up/out, quality control, and, consequently, production costs. The efficacy of the nanomaterials mentioned in this review has been demonstrated, as well as by means of in silico experiments, mostly in vitro on the opportune cancer cell lines and, in just a few cases, using in vivo experimental animals. Thus, the difficulty in extrapolating these experimental findings from isolated cell lines to complex biological systems and then to humans is evident. In the case of COVID-19, there is additional difficulty due to safety reasons. Studies of live SARS-CoV-2 are restricted to biosafety level 3 laboratories, so that SARS-CoV-2 pseudotyped viruses, with attenuated virulence in comparison with wild-type viruses, have been largely considered. However, they also have some limitations, especially concerning the extrapolation of findings to the real biological environment [301]. Furthermore, there are few robust small animal models showing phylogenetic differences in ACE2. Moreover, several genetic engineering rodent models have been developed, although they are not always valuable for mimicking the human SARS-CoV-2 disease [302].

At the current state of research, there is more and more numerous and significant evidence of the problems relating to the impact of nanomaterials on the health of individuals and the environment [303]. As to individuals’ health, nanotoxicity concerns are related to nanomaterial composition, dimension, and shape, as well as duration of exposure, penetration routes, biodistribution, tissue accumulation, and body clearence. Apart from the problems related to systemic toxicity, this aspect is of absolute importance even when the nanomaterial is used via inhalation [304,305]. Safety issues have to be taken into consideration not only in the case of therapeutic systems, but also in the case of nanosytems aimed at the development of products useful as viral spread control measures (from personal equipment to disinfectants and surface coatings) [43]. NPs, eventually released by these systems, can come into contact with individuals, producing toxic effects and also increasing environmental pollution.

Overall, it can be said that nanoscale technologies have provided promising results which still need to be evaluated for efficacy and safety.

## Data Availability

Data are available on request.

## Supplementary Materials

The following supporting information can be downloaded at: https://www.mdpi.com/article/10.3390/biom12081060/s1, Table S1: Autodock-estimated free energies of binding to SBD and NBD (kcal/mol); Figure S1: Atomic root mean square deviations of best scoring ligands, in complex with GRP78 SBD, during 48 ns of MD simulation; Figure S2: Molecular dynamics simulation results on 3-benzoylhosloppone; Figure S3: Molecular dynamics simulation results on masticadienoic acid; Figure S4: Molecular dynamics simulation results on isoiguesterin; Figure S5: Molecular dynamics simulation results on digitoxin; Figure S6: Molecular dynamics simulation results on glycyrrhizin; Figure S7: Molecular dynamics simulation results on 3-O-β-D-glucuronosyl-glycyrrhetinic acid; Figure S8: Chemical structure of the 24 considered compounds.

## Abbreviations

The following abbreviations are used in this manuscript:

ACE2	Angiotensin converting enzyme 2.
ADME	Absorption, Distribution, Metabolism, and Excretion.
aPDT	Antimicrobial photodynamic therapy.
ARDS	Acute respiratory distress syndrome.
ATP	Adenosine triphosphate.
AZT	Azithromycin.
BER	Berberin.
BSA	Bovine serum albumin.
BTB	Baricitinib.
CNP	3-cyanopyridine.
Cys	Cysteine.
COVID	Coronavirus disease.
CT	Chitosan.
CTAB	Cetyltrimethylammonium bromide.
DHEA	Dehydroepiandrosterone.
DDSs	Drug delivery systems.
DNICs	Dinitrosyl iron complexes.
EPA	Environmental Protection Agency.
ER	Endoplasmic reticulum.
EUA	Emergency Use Authorization.
FDA	Food and Drug Administration.
Fe${}_{m}$O${}_{n}$ NPs	iron oxide nanoparticles.
GCPQ	N-palmitoyl-N-monomethyl-N,N-dimethyl-N,N,N-trimethyl-6-O-glycolchitosan.
GLU	Glutamate–urea-based ligand.
GO	Graphene oxide.
H2@C60	Steroid–endohedral fullerene.
HEK	Human embryonic kidney.
His	Histidine.
HPVs	Human papillomaviruses.
HTCC	N-(2- hydroxypropyl)-3-trimethylammonium chitosan chloride.
IVM	Ivermectin.
LPH	Lipid polymer hybrid.
MERS	Middle-East Respiratory Syndrome.
MXenes	Transition metal carbides/nitrides.
Mpro	Main protease.
NCs	Nanocatchers.
NPs	Nanoparticles.
PDDA	Poly-diallyl-(dimethylammonium) chloride.
PLGA	Poly(lactic-co-glycolic acid).
PLpro	Papain-like protease.
PP	Polypropylene.
PSS	Poly (sodium 4-styrenesulfonate).
RBC	Red blood cell.
RBD	Receptor-binding domain.
RdRp	RNA-dependent RNA polymerase.
ROS	Reactive oxygen species.
RSV	Respiratory syncytial virus.
SARS-CoV-2	Severe Acute Respiratory Syndrome Coronavirus 2.
sgRNAs	Subgenomic RNAs.
SWCNTs	Single-walled carbon nanotubes.
Xenes	Metal carbides/nitrides.
3CLpro	3C-like proteinase.
