# Supplementary material for: Nanoscale Technologies in the Fight against COVID-19: From Innovative Nanomaterials to Computer-Aided Discovery of Potential Antiviral Plant-Derived Drugs
Source: Biomolecules. 2022 Jul 30;12(8):1060. doi: 10.3390/biom12081060 (PMC9405735; doi:10.3390/biom12081060)
Supplement: Supplementary file 1 [file biomolecules-12-01060-s001.zip › biomolecules-1804222-supplementary.pdf]

Supplementary Materials: Nanoscale technologies in the fight against COVID-19: from innovative nanomaterials to computer-aided discovery of potential antiviral plant-derived drugs

Nunzio Iraci, Carmelo Corsaro*, Salvatore V. Giofrè, Giulia Neri, Angela Maria Mezzasalma, Martina Vacalebre, Antonio Speciale, Antonina Saija, Francesco Cimino*, and Enza Fazio

1. In silico studies on terpenoids interaction with GRP78 protein

As reported in the main text, we investigated in silico 24 compounds as potential inhibitors of GRP78/spike interaction using the Autodock software on both NBD and SBD. Molecular docking simulations were performed on the structure of GRP78 (PDB ID:5E84), centering the two docking grids on the experimental bound conformation of ATP for the NBD and on residues Ile426, Thr428, Val429, Val432, Thr434, Phe451, Ser452, Val457 and Ile459 for the SBD, according to the study by Ibrahim et al. [60]. Docking simulations at the NBD did not result in any favourable predicted binding affinity, ranging between -5.1 and
1.3 kcal/mol. Based on these results, no further investigation was carried on NBD. On the opposite, encouraging results were obtained from docking on GRP78 SBD as shown in the following Table S1.

Table S1. Autodock-estimated free energies of binding to SBD and NBD (kcal/mol).

	SBD	NBD
11a-dehydroxyisoterreulactone-A	-5.95	-3.69
24-methylenecycloartenol	-5.36	-3.52
3-benzoylhosloppone	-6.43	-4.22
3-O-β-D-glucuronosyl-glycyrrhetinic acid	-6.36	-4.59
carvacrol	-4.57	-3.37
cucurbitacin-B	-5.88	-3.21
deslanoside	-3.85	-1.37
digitoxin	-7.15	-2.38
digoxin	-5.91	-2.59
geraniol	-3.67	-2.98
glycyrrhetinic acid	-5.84	-4.62
glycyrrhizin	-6.24	-3.96
isoguesterin	-6.38	-4.5
L-4-terpineol	-4.58	-3.45
licorice-saponin-A3	-4.78	-2.62
limonene	-3.89	-3.04
masticadienoic acid	-6.87	-5.1
NPACT01552	-1.16	1.95
NPACT01557	-2.96	1.3
phytolaccoside-B	-4.97	-2.76
pinene	-4.68	-3.59
rediocide-A	-5.23	-1.17
sabinene	-3.84	-3.01
uncaric acid	-5.62	-4.05

Figure S1 shows the comparison among values of the atomic root mean square deviations (RMSD) for chosen ligands while figures S2-S7 report the corresponding individual root mean square fluctuations (RMSF) during the 48 ns long MD.

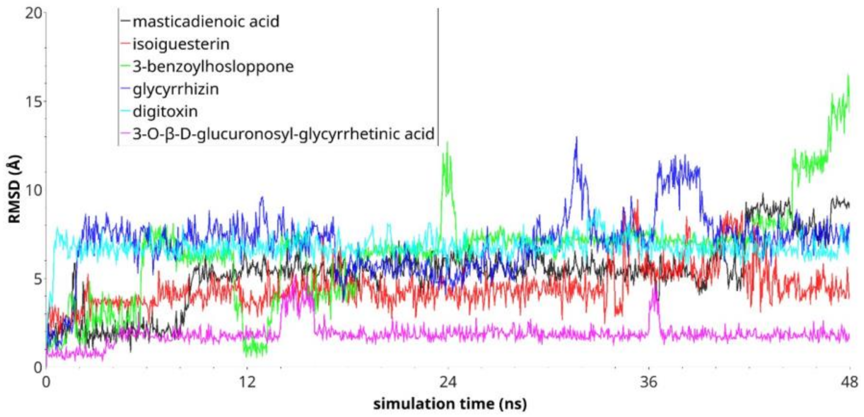

Figure S1. Atomic root mean square deviations of best scoring ligands, in complex with GRP78 SBD, during 48 ns of MD simulation.

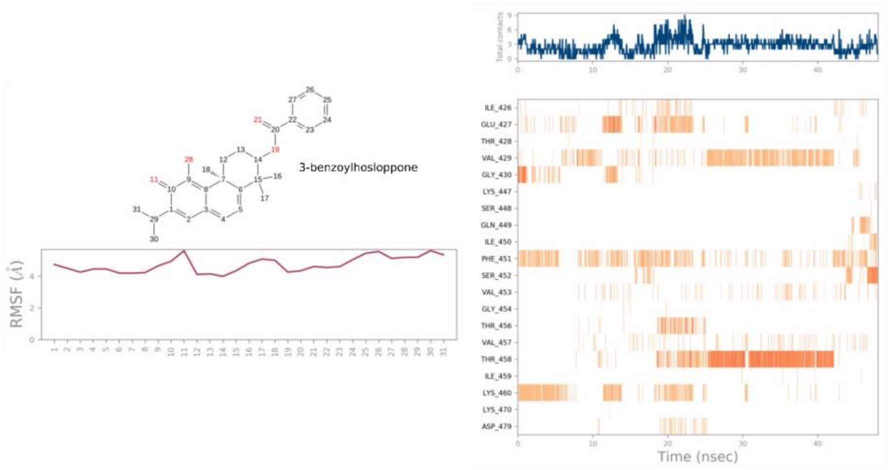

Figure S2. Left) Root mean square fluctuations of 3-benzoylhosloppone heavy atoms during the 48 ns long MD simulation in complex with GRP78. Right) Number of protein/ligand contacts as a function of MD simulation time (top) and residues interacting with the ligand for each trajectory frame (bottom).

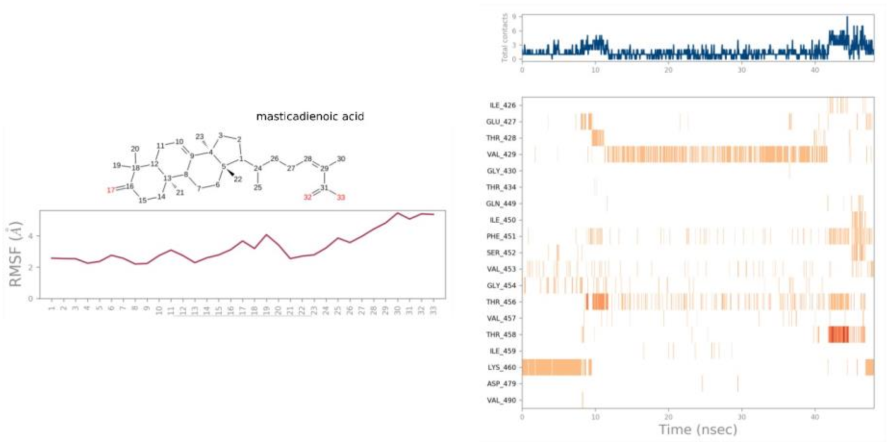

Figure S3. Left) Root mean square fluctuations of masticadienoic acid heavy atoms during the 48 ns long MD simulation in complex with GRP78. Right) Number of protein/ligand contacts as a function of MD simulation time (top) and residues interacting with the ligand for each trajectory frame (bottom).

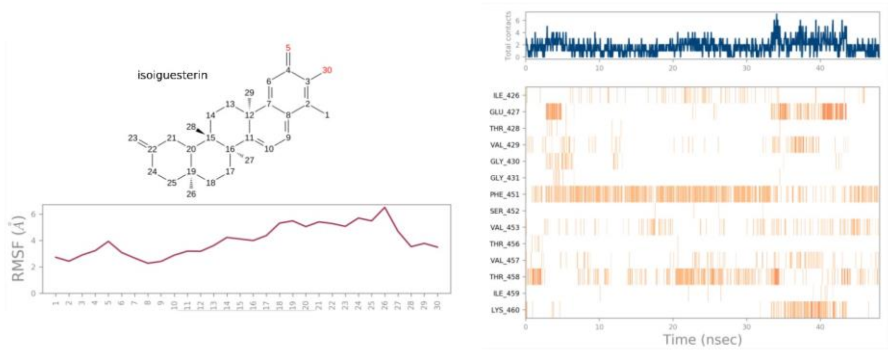

Figure S4. Left) Root mean square fluctuations of isoiguesterin heavy atoms during the 48 ns long MD simulation in complex with GRP78. Right) Number of protein/ligand contacts as a function of MD simulation time (top) and residues interacting with the ligand for each trajectory frame (bottom).

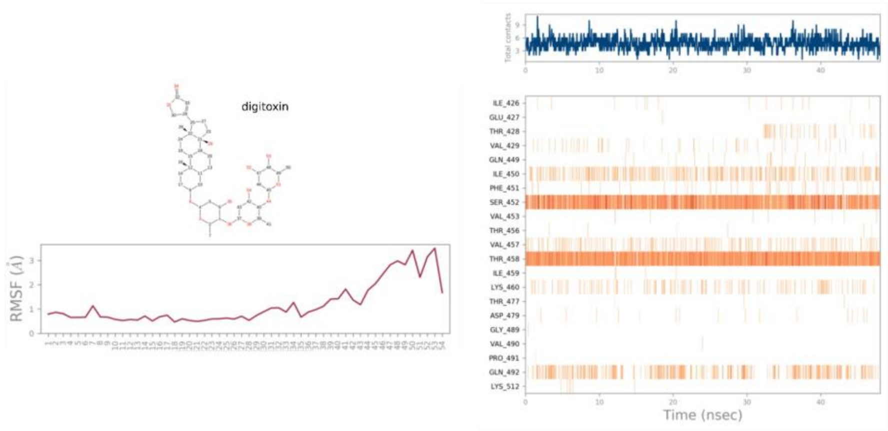

Figure S5. Left) Root mean square fluctuations of digitoxin heavy atoms during the 48 ns long MD simulation in complex with GRP78. Right) Number of protein/ligand contacts as a function of MD simulation time (top) and residues interacting with the ligand for each trajectory frame (bottom).

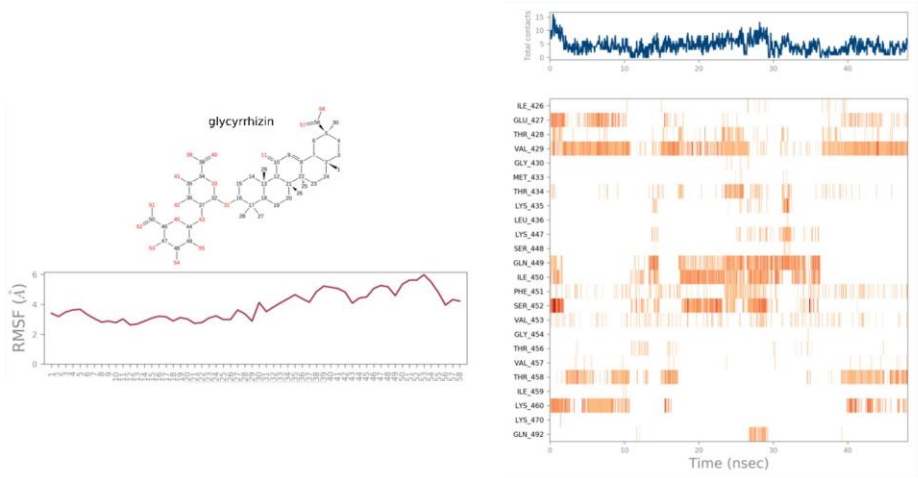

Figure S6. Left) Root mean square fluctuations of glycyrrhizin heavy atoms during the 48 ns long MD simulation in complex with GRP78. Right) Number of protein/ligand contacts as a function of MD simulation time (top) and residues interacting with the ligand for each trajectory frame (bottom).

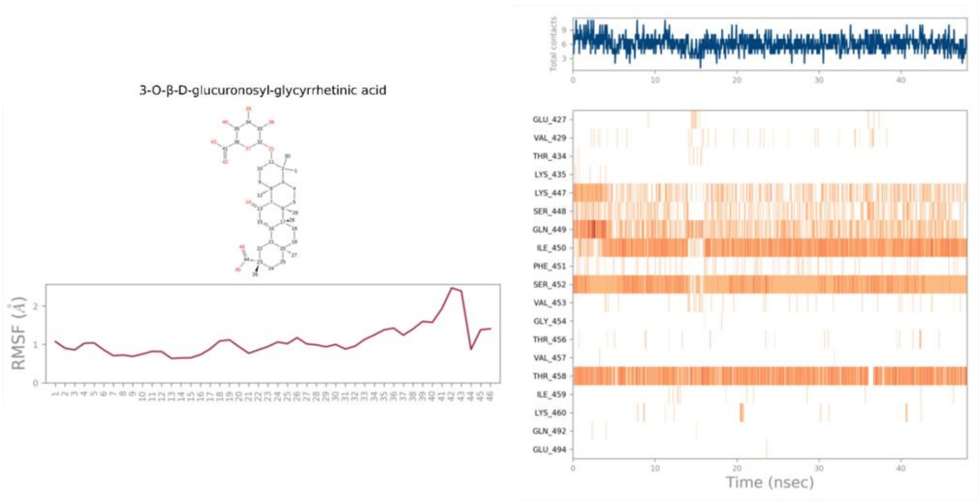

Figure S7. Left) Root mean square fluctuations of 3-O-β-D-glucuronosyl-glycyrrhetinic acid heavy atoms during the 48 ns long MD simulation in complex with GRP78. Right) Number of protein/ligand contacts as a function of MD simulation time (top) and residues interacting with the ligand for each trajectory frame (bottom).

2. Materials and methods for in silico studies

Ligand structures were retrieved from pubchem [292] while the docking target structure (PDB ID: 5E84 [293] was retrieved from the Protein Data Bank [294]. Protein and ligands were prepared for docking simulations by means of AutoDockTools 1.5.6. Two docking grids were generated through AutoGrid 4.2.6, i.e. the NBD and the SBD grids. For the NBD, the grid was centered on the ATP experimental bound conformation and its x, y and z dimensions were set to 70 points with a 0.375 Ågrid spacing. For the SBD, the grid was defined as a cubic box, centered on the centroid of residues Ile426, Thr428, Val429, Val432, Thr434, Phe451, Ser452, Val457 and Ile459, and its x, y and z dimensions were set to 80 points with a 0.375 Ågrid spacing. Docking simulations were performed using AutoDock 4.2.6. For each ligand and grid, 500 Lamarckian Genetic Algorithm runs, with 150 individuals and 2.5×10^5 energy evaluations, were run. The six best scoring docking solutions on the SBD grid were then imported into the Schrödinger Maestro GUI and the system builder utility was used to build the environments for MD simulations. MD simulations were run in explicit solvent, using the TIP3P water model [295] in a periodic boundary conditions orthorhombic box. Systems were neutralized by Na^+ and Cl^- ions, which were added until a concentration of 0.15 M was reached. The default six-stages relaxation protocol distributed with Desmond [296] was run prior to the production stage, that was run for 48 ns at a temperature of 300 K in the NPT ensemble using a Nose-Hoover chain thermostat and a Martyna-Tobias-Klein barostat (1.01325 bar), applying a 1 kcal/mol harmonic constrain on the backbone heavy atoms. Time steps were set to 2 fs, 2fs and 6fs for bonded, near and far interactions, respectively. No SHAKE nor LINCS approximations were applied. Analysis of the simulations were carried out using the Simulation Interaction Diagram embedded in the Schrödinger Maestro molecular modelling interface (Schrödinger Release 2021-1: Maestro, Schrödinger, LLC, New York, NY, 2021).

For completeness, Fig. S8 reports the structure of the 24 compounds we have considered in this in silico investigation.

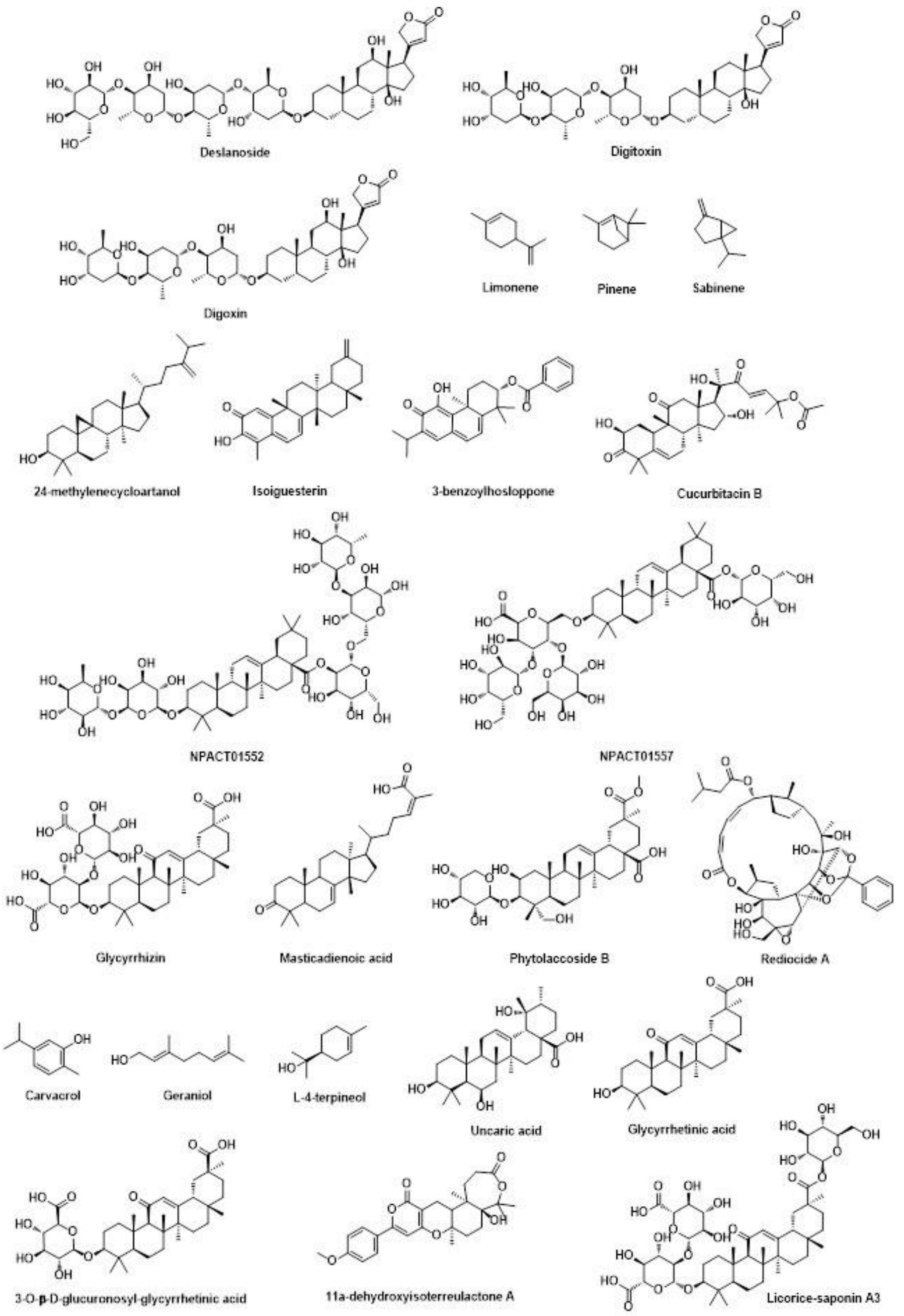

Figure S8. Chemical structure of the 24 considered compounds.
